# Transcriptional response of *Burkholderia cenocepacia *J2315 sessile cells to treatments with high doses of hydrogen peroxide and sodium hypochlorite

**DOI:** 10.1186/1471-2164-11-90

**Published:** 2010-02-05

**Authors:** Elke Peeters, Andrea Sass, Eshwar Mahenthiralingam, Hans Nelis, Tom Coenye

**Affiliations:** 1 Laboratory of Pharmaceutical Microbiology, Ghent University, Ghent, Belgium; 2 Cardiff School of Biosciences, Cardiff University, Cardiff, UK

## Abstract

**Background:**

*Burkholderia cepacia *complex bacteria are opportunistic pathogens, which can cause severe respiratory tract infections in patients with cystic fibrosis (CF). As treatment of infected CF patients is problematic, multiple preventive measures are taken to reduce the infection risk. Besides a stringent segregation policy to prevent patient-to-patient transmission, clinicians also advise patients to clean and disinfect their respiratory equipment on a regular basis. However, problems regarding the efficacy of several disinfection procedures for the removal and/or killing of *B. cepacia *complex bacteria have been reported. In order to unravel the molecular mechanisms involved in the resistance of biofilm-grown *Burkholderia cenocepacia *cells against high concentrations of reactive oxygen species (ROS), the present study focussed on the transcriptional response in sessile *B. cenocepacia *J2315 cells following exposure to high levels of H_2_O_2 _or NaOCl.

**Results:**

The exposure to H_2_O_2 _and NaOCl resulted in an upregulation of the transcription of 315 (4.4%) and 386 (5.4%) genes, respectively. Transcription of 185 (2.6%) and 331 (4.6%) genes was decreased in response to the respective treatments. Many of the upregulated genes in the NaOCl- and H_2_O_2_-treated biofilms are involved in oxidative stress as well as general stress response, emphasizing the importance of the efficient neutralization and scavenging of ROS. In addition, multiple upregulated genes encode proteins that are necessary to repair ROS-induced cellular damage. Unexpectedly, a prolonged treatment with H_2_O_2 _also resulted in an increased transcription of multiple phage-related genes. A closer inspection of hybridisation signals obtained with probes targeting intergenic regions led to the identification of a putative 6S RNA.

**Conclusion:**

Our results reveal that the transcription of a large fraction of *B. cenocepacia *J2315 genes is altered upon exposure of sessile cells to ROS. These observations have highlighted that *B. cenocepacia *may alter several pathways in response to exposure to ROS and they have led to the identification of many genes not previously implicated in the stress response of this pathogen.

## Background

The *Burkholderia cepacia *complex is a group of Gram-negative β-proteobacteria that comprises at least 17 species [1]. Originally identified as plant pathogens [2], these versatile microorganisms have emerged as notorious pathogens in cystic fibrosis (CF) patients [3]. One of the best-studied aspects of these opportunistic pathogens is their high level of resistance to a broad range of antimicrobial agents. Due to this multi-drug resistance, treatment of *B. cepacia *complex infected CF patients is problematic [4] and a *B. cepacia *complex positive status is often associated with increased morbidity and mortality [5].

As several outbreaks with genetically distinct *B. cepacia *complex strains have occurred, stringent infection control guidelines were brought into force; these guidelines not only aim to segregate infected patients from non-infected ones, but also emphasize the importance of good hand hygiene practices and frequent disinfection of environmental surfaces and respiratory equipment [6].

In a recent study, we showed that some of the current disinfection protocols are inadequate to effectively remove and kill biofilm-grown *Burkholderia cenocepacia *cells [7]. Biofilm formation has been described for several *B. cepacia *complex strains and is considered to be an important virulence trait [5]. The observation that these sessile cells are highly resistant against H_2_O_2 _and NaOCl is worrying since these oxidative agents are also very important in the endogenous defence against microorganisms. During infection, neutrophils normally produce a variety of reactive oxygen species (ROS), including superoxide, H_2_O_2 _and hypochlorite [8]. H_2_O_2 _and NaOCl can react with intracellular iron via the Fenton reaction and the resulting highly reactive hydroxyl radicals will damage lipids, proteins and DNA [9,10].

In addition, *B. cepacia *complex bacteria are one of the most common contaminants of pharmaceutical and industrial products [11] and this feature is probably highly dependent on their ability to form persistent biofilms.

The fact that *B. cenocepacia *cells can survive high doses of exogenous ROS, suggests that this microorganism possesses various protective mechanisms involved in the scavenging and neutralization of ROS. Thus far, the role of KatA and KatB in the survival of planktonic *B. cenocepacia *cells has been studied [12] and the presence of genes encoding catalases and alkyl hydroperoxide reductases within the *B. cenocepacia *J2315 genome has been described [13]. A recent transcriptomic study of *B. cenocepacia *planktonic growth in CF sputum, an environment that is rich in ROS, demonstrated that an organic hydroperoxide resistance gene (BCAL2753) and an OsmC-like oxidative stress gene (BCAL1766) were highly upregulated [14]. However, no data are available yet regarding the wider molecular mechanisms used by sessile *B. cenocepacia *cells to survive acute exposure to H_2_O_2 _or NaOCl. In view of the large fractions of sessile cells that can survive high levels of ROS, a better insight into the genomic basis of this major virulence trait is required.

Over the last decade, many studies have been performed on the molecular mechanisms involved in bacterial resistance against ROS. These studies include microarray analyses in which the transcriptional response in untreated planktonic cells was compared with the transcriptional response in ROS-exposed planktonic cells [15-21]. In addition, the effect of various mutations on the survival of bacteria in the presence of ROS and oxygen has been evaluated in a number of other studies [22-28]. Although the biofilm mode of growth is the prevailing microbial lifestyle [29], no microarray analyses focussing on the transcriptional response of sessile cells exposed to ROS have been performed thus far. In the present study, we compared gene expression in treated and untreated *B. cenocepacia *J2315 biofilms, using microarray analysis and qPCR. In addition, the role of several genes was further examined using mutant strains.

## Results and discussion

### Treatment of *B. cenocepacia *J2315 biofilms with H_2_O_2 _and NaOCl

**Figure 1:**
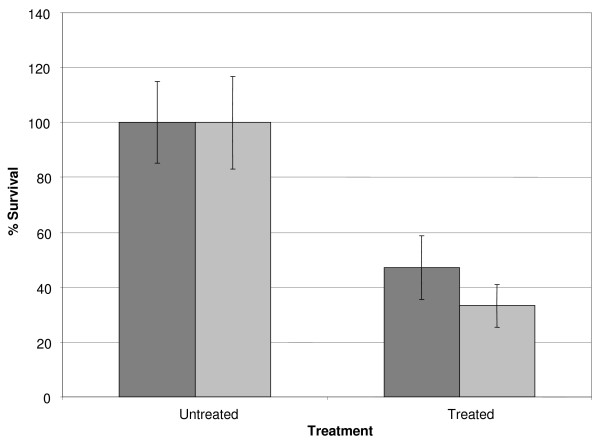
**Effect of treatments with H**_**2**_**O**_**2 **_**or NaOCl on the number of viable sessile *B. cenocepacia *J2315 cells**. The average relative fluorescence signals (%) show the fraction of viable *B. cenocepacia *J2315 sessile cells in untreated biofilms and in biofilms treated with H_2_O_2 _(0.3%, 30 min; dark grey bars) or NaOCl (0.02%, 5 min; pale grey bars). Error bars represent standard deviations.

Previous research indicated that disinfection protocols based on the use of H_2_O_2 _(3%, 30 min) and NaOCl (0.05%, 5 min) (as recommended by several CF patient organisations) have insufficient activity against *B. cenocepacia *biofilms [7]. Based on these disinfection protocols, we determined the H_2_O_2_- and NaOCl-concentrations (using similar treatment times) that resulted in a reduction in the number of viable cells of approximately 50% compared to untreated biofilms. Using this approach, we ensured that a sufficient number of viable cells would remain as a source of mRNA for microarray analyses. A resazurin based viability staining performed on treated and untreated *B. cenocepacia *J2315 cells revealed that exposure to 0.3% H_2_O_2 _(30 min) or 0.02% NaOCl (5 min) killed approximately half the sessile *B. cenocepacia *J2315 population (Figure 1).

### Transcriptomic response to high levels of oxidative stress: microarray data analysis

Compared to the normalized signal intensities obtained for the untreated *B. cenocepacia *J2315 sessile cells, the normalized signal intensity for 618 (6.0%) and 854 (8.3%) probes was significantly altered after treatments with H_2_O_2 _and NaOCl, respectively (Table 1). In response to the treatment with H_2_O_2_, using a fold change cut off of 2 (p < 0.05), the expression of 315 (4.4%) genes was significantly higher (Additional file 1), while the expression of 185 (2.6%) genes was lower (Additional file 2). Using the same data analysis parameters, treatment of the biofilms with 0.02% NaOCl resulted in a significant upregulation of 386 (5.4%) genes (Additional file 3) and downregulation of 331 (4.6%) genes (Additional file 4). A total of 171 (2.4%) and 117 (1.6%) genes showed a higher or lower expression in response to both treatments. The fraction of up- and downregulated genes is comparable to the fraction of genes showing a significantly altered expression in planktonic *Pseudomonas aeruginosa *PAO1 cells exposed to sublethal concentrations of H_2_O_2 _or NaOCl (fold change >2; p < 0.05) [21].

**Table 1:** Overview of the number (fraction) of probes for which a significant up- or downregulation was observed in response to treatments with H_2_O_2 _or NaOCl.

	**H**_**2**_**O**_**2 **_**(0.3%, 30 min)**	NaOCl (0.02%, 5 min)
*Upregulated*		

CDS (n = 7153)	315 (4.4%)	386 (5.4%)
IG (n = 1520)	39 (2.6%)	56 (3.7%)
tRNA (n = 47)	20 (42.6%)	8 (17.0%)
rRNA (n = 9)	9 (66.7%)	1 (11.1%)

*Downregulated*		

CDS (n = 7153)	185 (2.6%)	331 (4.6%)
IG (n = 1520)	54 (3.6%)	68 (4.5%)
tRNA (n = 47)	0 (0%)	3 (6.4%)
rRNA (n = 9)	0 (0%)	0 (0%)

The number of probes on the array that correspond to *B. cenocepacia *J2315 protein encoding sequences (CDS), intergenic regions (IGs) and tRNA- or rRNA-encoding sequences are shown for each category (n).

Among the upregulated genes, many were known to prevent, counteract or repair damage resulting from oxidative stress. Furthermore, the expression of various genes involved in the synthesis and the assembly of the flagellum was also increased. In addition to the changes in expression of a variety of protein-encoding genes and tRNA-encoding sequences, a change in expression was observed for many intergenic regions (IGs), which might contain non-coding regulatory RNA (ncRNA). The transcription of several genes belonging to a BcepMu prophage was also significantly increased in biofilms exposed to H_2_O_2 _for 30 or 60 min. These gene sets with significantly altered expression are discussed in more detail below.

### Genes involved in protection against oxidative stress

**Figure 2:**
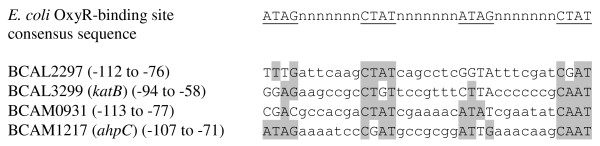
**Putative OxyR-binding sites upstream of BCAL2297, BCAL3299 (*katB*), BCAM0931 and *ahpCF *(BCAM1217-BCAM1216)**. The four tetranucleotide sequences in the *E. coli *OxyR-binding consensus sequence are underlined and the nucleotides matching the consensus sequence are indicated by grey shading [74].

A large proportion of the upregulated genes were known to be involved in the protection against oxidative stress (Table 2). In other Gram-negative microorganisms such as *Escherichia coli *and *P. aeruginosa*, coordinated regulation of many genes associated with oxidative stress is mediated by the transcriptional regulator OxyR [15,26]. In *E. coli*, oxidation of OxyR leads to an increased expression of various genes, including genes coding for alkyl hydroperoxide reductases C and F (*ahpCF*; *B. cenocepacia *homologues: BCAM1217 and BCAM1216) and hydroperoxidase I (*katG*; homologue: BCAL3299 [annotated as *KatB*]) [15]. In *P. aeruginosa*, oxidized OxyR increases the expression of *ahpCF *and *katB *(homologue: BCAM0931) [26]. A putative OxyR-binding site was found upstream of all genes encoding homologues of the latter proteins in *B. cenocepacia *J2315 (*katB*, BCAM0931 and *ahpCF*; Figure 2). Furthermore, a putative OxyR box was also found upstream of the highly upregulated gene BCAL2297, which encodes a hypothetical protein (Figure 2).

**Table 2:** List of genes involved in the (oxidative) stress response of sessile *B. cenocepacia *J2315 cells exposed to H_2_O_2 _or NaOCl.

		Fold change in expression in treated biofilms vs untreated biofilms				
**Gene number**	**Annotation**	**Microarray: H**_**2**_**O**_**2 **_**(30 min)**	**qPCR: H**_**2**_**O**_**2 **_**(15 min)**	**qPCR: H**_**2**_**O**_**2 **_**(30 min)**	**qPCR: H**_**2**_**O**_**2 **_**(60 min)**	**Microarray: NaOCl (5 min)**

*Genes involved in the oxidative stress response*						

BCAS0085	Organic hydroperoxide resistance protein	49.3	1438.4	1307.0	560.7	20.3
BCAM1217	Alkyl hydroperoxide reductase C subunit (*ahpC*)	41.3	68.7	46.7	20.4	15.0
BCAL0771	Non-heme chloroperoxidase	37.7	27.0	65.8	97.5	NS
BCAM0896	Organic hydroperoxide resistance protein	36.5	27.6	23.2	15.1	37.2
BCAL1766	OsmC-like protein (Ohr protein, see text)	18.0	28.2	43.8	36.9	75.2
BCAM1216	Alkyl hydroperoxide reductase F subunit (*ahpF*)	15.5	182.5	122.5	49.5	2.1
BCAM0931	Catalase precursor	5.7	98.8	32.3	6.1	NS
BCAL1106	Cytochrome b561 family protein	4.1	1.7	12.7	42.5	8.3
BCAL2780	Putative thioredoxin protein	2.9	1.9	3.1	3.3	NS
BCAL3035	Thioredoxin reductase (*trxB*)	2.8	-	-	-	NS
BCAM0930	Putative ankyrin-like protein (*ankB*)	2.7	-	-	-	NS
BCAL3477	Putative catalase	2.7	1.7	6.5	23.1	NS
BCAL2014	Carboxymuconolactone decarboxylase family	2.7	2.3	2.1	1.7	3.3
BCAL1761	MarR family regulatory protein (*ohrR*)	2.4	-	-	-	NS
BCAL2013	AhpC/TSA family protein	2.1	-	-	-	NS
BCAL1900	Thioredoxin A (*trxA*)	2.0	-	-	-	2.1
BCAL3299	Catalase (*katB*)	1.8	1.4	1.7	NS	NS
BCAM2107	Catalase (*katA*)	NS	NS	NS	NS	NS
BCAL3214	Carboxymuconolactone decarboxylase family	NS	-	-	-	35.1

*Genes involved in protection and repair of DNA*						

BCAL0953	Putative recombinase A (*recA*)	2.6	NS	1.8	NS	1.9
BCAL3297	DNA-binding protein (*dps*)	NS	-	-	-	NS

*Genes involved in the repair of iron-sulfur clusters and iron-sulfur cluster containing proteins*						

BCAM0961	Aconitase A (*acnA*)	NS	-	-	-	NS
BCAM1833	Aconitase B (*acnB*)	5.1	2.7	3.5	3.1	2.1
BCAL2192	Conserved hypothetical protein (*iscX*)	2.4	-	-	-	2.4
BCAL2193	Ferredoxin (*fdx*)	2.5	-	-	-	2.5
BCAL2194	Chaperone protein HscA homologue (*hscA*)	2.4	-	-	-	2.4
BCAL2195	Chaperone protein HscB homologue (*hscB*)	2.3	-	-	-	2.3
BCAL2196	Iron-sulfur cluster assembly protein (*iscA*)	1.6	-	-	-	1.6
BCAL2197	Iron-sulfur cluster scaffold protein (*iscU*)	3.4	-	-	-	3.4
BCAL2198	Cysteine desulfurase (*iscS*)	2.9	-	-	-	2.9
BCAL2199	Transcriptional regulator (*iscR*)	2.3	-	-	-	2.3

*Sigma factors*						

BCAL0787	RNA polymerase σ^32 ^factor	4.7	2.3	2.7	2.5	2.6
BCAL1369	Putative RNA polymerase σ^70 ^factor (*fecI*)	2.2	-	-	-	NS
BCAL1688	Putative RNA polymerase σ^70 ^factor (*orbS*)	6.4	5.7	5.9	12.9	NS
BCAL3478	Putative RNA polymerase σ factor	3.4	NS	5.5	16.6	NS

The results represent the fold change in gene expression in the treated biofilms compared to the gene expression in the untreated biofilms. -: no qPCR experiments were performed. NS: no significant change in expression was observed between the treated and the untreated biofilms (p > 0.05).

Although the transcription of both *ahpCF *(BCAM1217-BCAM1216) and BCAM0931 was markedly increased after treatment with H_2_O_2 _(Table 2), the increased transcription of BCAL3299 (*katB*) was less pronounced: the microarray data revealed a statistically significant 1.8-fold upregulation following treatment with H_2_O_2_, which was confirmed by qPCR (Table 2). The importance of the AhpC protein in the protection against oxidative stress has been reported previously [30] and the high upregulation of both *ahpC *and *ahpF *confirms these data. Yet, the limited upregulation of *katB *was surprising, as KatB is supposed to be the major catalase/peroxidase in *B. cenocepacia *and hypersensitivity of planktonic *B. cenocepacia *MDL2 (*katB *mutant) cells to H_2_O_2 _has been reported [12]. As expected, the expression of the minor catalase/peroxidase encoding gene *katA *(BCAM2107) was not altered in response to exogenous oxidative stress [12].

**Figure 3:**
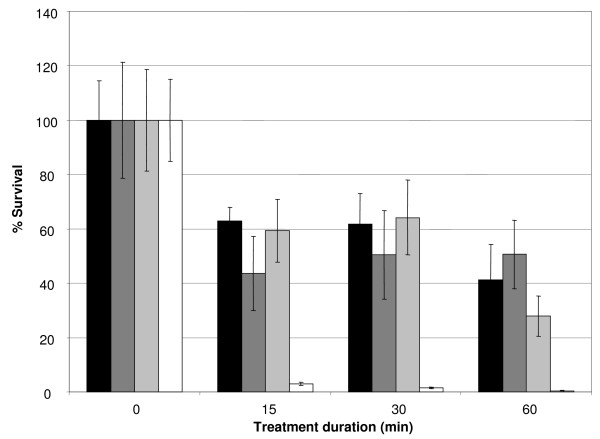
**Effect of treatment with H**_**2**_**O**_**2 **_**on the number of viable sessile *B. cenocepacia *cells**. The average relative fluorescence signals (%) show the fraction of viable sessile cells in untreated biofilms and in biofilms treated with H_2_O_2 _(0.3%) for 15, 30 or 60 min. Data were obtained for biofilms of *B. cenocepacia *J2315 (black bars), C5424 (dark grey bars), MDL1 (*katA *mutant; pale grey bars) and MDL2 (*katB *mutant; white bars). Error bars represent standard deviations.

In order to further clarify the importance of KatB for the survival of *B. cenocepacia *biofilms exposed to H_2_O_2_, biofilms of two wild type strains (*B. cenocepacia *C5424 and J2315) and of two mutant strains (*B. cenocepacia *MDL1 [*katA *mutant] and MDL2 [*katB *mutant]) were treated with 0.3% H_2_O_2 _and the fraction of surviving sessile cells was determined using a resazurin based viability staining [31]. In addition, the remaining fraction of H_2_O_2 _was determined in the supernatants of these biofilms after treating them for 15, 30 or 60 min. It should be noted that strains J2315 and C5424 are not identical, although they both belong to the ET12 lineage; it is at present unclear whether this has an impact on our results. These results show that, in contrast to the observations for the wild type strains and for the *katA *mutant strain, less than 5% and 0.5% of all sessile cells present in the *B. cenocepacia katB *mutant biofilms survived 15 and 60 min treatments with 0.3% H_2_O_2_, respectively (Figure 3). In addition, approximately 90% of all initially added H_2_O_2 _was still present in the supernatant of the *B. cenocepacia katB *mutant biofilms, whereas only trace amounts of H_2_O_2 _were recovered in the supernatants of the biofilms of the wild type strains and of the *katA *mutant (data not shown). In keeping with these observations, strong effervescence was observed when H_2_O_2 _was added to the biofilms of both wild type strains and of the *katA *mutant, whereas no effervescence was observed for the *B. cenocepacia katB *mutant biofilms. The data obtained in these experiments clearly indicate that KatB is not only essential for the survival of planktonic *B. cenocepacia *cells exposed to H_2_O_2_, but that this catalase/peroxidase is also crucial for the protection of sessile *B. cenocepacia *cells against exogenous H_2_O_2_. Conforming with previous data for stationary phase planktonic cultures [12], we observed a substantial expression of *katB *in untreated biofilms, which suggests the involvement of other factors in the regulation of the expression of this particular gene during sessile growth. The transcriptional regulation by various transcription factors might also explain the limited upregulation of *katB *under conditions of oxidative stress [32].

In addition to the upregulation of *katB *by these sessile populations, an increased transcription of two other catalase encoding genes (BCAM0931 and BCAL3477) was observed in the H_2_O_2_-treated biofilms. These two catalase encoding genes possessed differential expression patterns in relation to the length of H_2_O_2 _exposure: while BCAM0931 showed the highest upregulation in the biofilms treated for 15 min, the transcription of BCAL3477 was much more pronounced in the 60 min treated biofilms (Table 2). As only trace amounts of H_2_O_2 _were still present after a 15 min treatment, the increased transcription of BCAL3477 in biofilms treated for longer periods suggests that it does not participate in the direct neutralization of exogenous H_2_O_2 _and that the expression of this catalase is triggered indirectly. We also observed that the increased transcription of BCAM0931 was accompanied by the increased expression of its flanking gene *ankB *(BCAM0930; Table 3). In *P. aeruginosa*, these two genes are part of a small operon and the lack of a functional AnkB protein results in a decreased KatB activity and an increased sensitivity to H_2_O_2 _[25].

**Table 3:** Clusters of adjacent genes that show similar changes in expression when *B. cenocepacia *J2315 biofilms are exposed to H_2_O_2 _or NaOCl.

		Fold change in expression in treated biofilms vs untreated biofilms	
**Gene number**	**Annotation**	**H**_**2**_**O**_**2 **_**(30 min)**	**NaOCl (5 min)**

BCAL1763	Putative exported protein	15.5	30.2
BCAL1764	Putative exported protein	5.3	15.3
BCAL1765	Putative exported protein	7.5	31.0
BCAL1766	OsmC-like protein (Ohr protein, see text)	18.0	75.2

BCAS0084	TetR family regulatory protein	5.9	3.8
BCAS0085	Organic hydroperoxide resistance protein	49.3	20.3
BCAS0086	Putative exported lipase	96.6	11.3

BCAM0930	Putative ankyrin-like protein (*ankB*)	2.7	NS
BCAM0931	Catalase precursor	5.7	NS

BCAM1216	Alkyl hydroperoxide reductase F subunit (*ahpF*)	15.5	2.1
BCAM1217	Alkyl hydroperoxide reductase C subunit (*ahpC*)	41.3	15.0

BCAL1688	Putative RNA polymerase σ ^70 ^factor (*orbS*)	6.4	NS
IG1_1847123	IG between BCAL1688 and BCAL1689	2.9	NS
BCAL1689	Mbt-like protein (*orbH*)	3.8	NS
BCAL1690	Dioxygenase (*orbG*)	2.3	NS

BCAL2192	Conserved hypothetical protein (*iscX*)	2.4	NS
BCAL2193	Ferredoxin (*fdx*)	2.5	NS
BCAL2194	Chaperone protein HscA homologue (*hscA*)	2.4	NS
BCAL2195	Chaperone protein HscB homologue (*hscB*)	2.3	NS
BCAL2196	Iron-sulfur cluster assembly protein (*iscA*)	1.6	NS
BCAL2197	Iron-sulfur cluster scaffold protein (*iscU*)	3.4	NS
BCAL2198	Cysteine desulfurase (*iscS*)	2.9	NS
BCAL2199	Transcriptional regulator protein (*iscR*)	2.3	NS

BCAL1105	Exported protein	6.8	12.7
BCAL1106	Cytochrome b561 family protein	4.1	8.3
BCAL1107	Oxidoreductase	2.7	3.4

BCAL3477	Putative catalase	2.7	NS
BCAL3478	Putative RNA polymerase σ factor	3.4	NS

The results represent the fold change in gene expression in the treated biofilms compared to the gene expression in the untreated biofilms (based on the microarray analysis). NS: no significant change in expression was observed between the treated and the untreated biofilms (p > 0.05).

In *E. coli *the OxyR regulon also comprises genes encoding glutaredoxin 1 (*grxA*) and a glutathione reductase (*gorA*) [33], yet no significant upregulation of *gorA *or of any of the glutaredoxin encoding genes was observed in the ROS exposed sessile *B. cenocepacia *J2315 cells. We did observe a significant upregulation of the expression of a thioredoxin reductase (TrxB; encoded by BCAL3035), of thioredoxin A (TrxA; encoded by BCAL1900) and of a putative thioredoxin (BCAL2780), although no OxyR-boxes are present upstream of these genes. Since thioredoxins are capable of reducing oxidized proteins and of scavenging hydroxyl radicals [34], their upregulated expression in H_2_O_2_-treated *B. cenocepacia *cells was not unexpected. In addition, the strong upregulation of a carboxymuconolactone decarboxylase encoding gene (BCAL3214) in response to NaOCl exposure and the significant upregulation of a second carboxymuconolactone decarboxylase encoding gene (BCAL2014) in response to exposure to both H_2_O_2 _and NaOCl, suggest that both these proteins are also involved in the neutralization of exogenous ROS in *B. cenocepacia *J2315 biofilms (Table 2).

**Figure 4:**
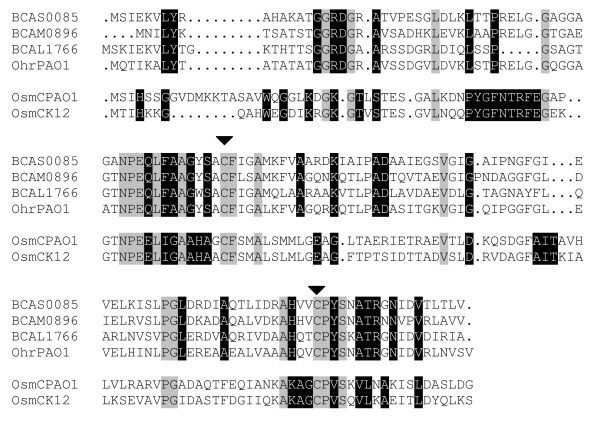
**The protein encoded by BCAL1766 belongs to the Ohr family**. Amino acid sequences of three upregulated Ohr proteins in *B. cenocepacia *J2315 (encoded by BCAS0085, BCAM0896 and BCAL1766) and of *P. aeruginosa *PAO1 Ohr (OhrPAO1) and OsmC (OsmCPAO1) and *E. coli *K-12 MG1655 OsmC (OsmCK12) were aligned using the CLUSTAL W program using standard settings [75]. The regions of black shading and white lettering show the conserved regions found in either the Ohr homologues or the OsmC homologues. Grey shading indicates that identical amino acid sequences are found in both Ohr and OsmC homologues. The triangles point to the highly conserved C residues in both families.

Treatments with H_2_O_2 _or NaOCl resulted in the increased transcription of three organic hydroperoxide resistance (*ohr*) genes: BCAS0085, BCAM0896 and BCAL1766. Although the latter gene encodes a protein that is annotated as an OsmC-like protein, the protein has the typical characteristics of the Ohr subfamily ([35]; Figure 4). Furthermore, BCAL1766 and BCAM0896 are both located in close proximity of putative OhrR encoding genes (BCAL1761 and BCAM0897 [43% and 53% identity to *Xanthomonas campestris *OhrR, respectively]). In a recent study by Drevinek et al. [14], the upregulation of a further *ohr *family gene, BCAM2753, was observed in planktonic cells exposed to sub-inhibitory concentrations of H_2_O_2 _and organic hydroperoxides; however, no such upregulation was observed in H_2_O_2_-treated sessile cells. Overall, such a strong upregulation of the expression of *ohr *genes in response to H_2_O_2 _or NaOCl treatments has not been reported thus far [36]. *Ohr *expression in *P. aeruginosa *PAO1 appears to be triggered specifically by organic hydroperoxides [26] and Ohr proteins preferably reduce organic, rather than inorganic hydroperoxides [37]. In addition to the upregulation of BCAS0085 and BCAL1766, similar expression patterns were observed for their neighbouring genes, suggesting that the genes in both clusters are co-transcribed (Table 3). Overall, our results suggest that Ohr proteins are very important for the protection of biofilm-grown *B. cenocepacia *cells against ROS and that it can draw on multiple genes associated with this family of stress proteins.

**Figure 5:**
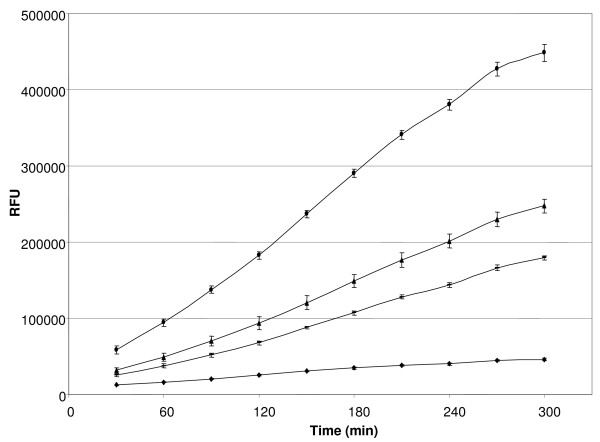
**Increased lipase activity in the supernatant of H**_**2**_**O**_**2**_**-treated *B. cenocepacia *J2315 biofilms**. Lipase activity was determined in the supernatant of *B. cenocepacia *J2315 biofilms that were either untreated (diamonds) or that were treated with 0.3% H_2_O_2 _for 15 (rectangles), 30 (triangles) or 60 min (circles). The graph shows a representative example of the obtained curves using 4-MU oleate as fluorogenic substrate. Signals are expressed as normalized fluorescence units (RFU). Error bars represent standard deviations.

In addition to the upregulation of BCAS0085, we also observed a marked increase in the expression of BCAS0086, which encodes an exported lipase. This overexpression is probably the result of the cotranscription of this gene with BCAS0085. In order to determine whether this overexpression results in an increased extracellular lipase activity, the cleavage of three fluorogenic 4-methylumbelliferyl substrates (4-MU palmitate, 4-MU stearate and 4-MU oleate) was determined in the supernatants of treated and untreated biofilms [38]. For all substrates used, the fluorescent signal was higher in the treated biofilms compared to the untreated biofilms, confirming the increased extracellular lipase activity for the treated biofilms (Figure 5). Within the genomes of the sequenced *B. cepacia *complex strains, orthologues of BCAS0085 and BCAS0086 are organised in a similar operon-like fashion and therefore we examined the induction of lipase expression by H_2_O_2 _in biofilms of six *B. cepacia *complex reference strains. For *B. cenocepacia *C5424, HI2424, *Burkholderia ambifaria *LMG 19182 and *Burkholderia dolosa *AU0158, an increased lipase activity was observed in the supernatant of all treated biofilms. Similarly, lipase activity was increased in the supernatant of *B. cenocepacia *AU1054 biofilms treated for 30 and 60 min, and in the supernatant of a 60 min treated *Burkholderia multivorans *LMG 17588 biofilm (data not shown). These data confirmed that lipase activity in the supernatant of *B. cepacia *complex biofilms following exposure to oxidative stress is increased, which is probably due to the co-transcription of BCAS0086 orthologues with BCAS0085 orthologues.

The transcription of a cytochrome b561 encoding gene (BCAL1106) and of its adjacent genes (BCAL1105 and BCAL1107) was also highly increased when the biofilms were exposed to exogenous ROS (Table 3). The qPCR data clearly indicate that prolonged exposure to H_2_O_2 _results in an increasing overexpression of BCAL1106 (Table 2). Yet, even after a 5 min exposure to NaOCl, a considerable overexpression of all three genes was observed (Table 3). Drevinek et al. [14] recently reported a high upregulation of these genes in *B. cenocepacia *J2315 cells grown in CF sputum and they suggested that this may result from the presence of ROS in CF sputum. The microarray data also revealed that the transcription of BCAL0771, a gene encoding a non-heme chloroperoxidase increased drastically in biofilms exposed to H_2_O_2_. This finding was confirmed by qPCR and an increased expression was observed in response to prolonged treatments (Table 2). Although four genes encoding a non-heme chloroperoxidase are present in the *B. cenocepacia *J2315 genome (BCAL0771, BCAM2106, BCAM2109, BCAS0079), overexpression was only observed for BCAL0771, indicating the ability of our microarray-based strategy to highlight the most active gene in *Burkholderia *genomes where multiple paralogous functional pathways exist [13].

### Upregulation of *recA*

H_2_O_2 _and NaOCl can both damage nucleic acids. In the Fenton reaction, H_2_O_2 _reacts with Fe^2+ ^resulting in the formation of highly reactive hydroxyl radicals [9]. Similarly, NaOCl can also generate hydroxyl radicals via a Fenton-type reaction [10]. Because positively charged Fe^2+ ^ions are often localized along the negatively charged phosphodiester backbone of nucleic acids, DNA is a vulnerable target for harmful reactions with these radicals [39]. As exposure to ROS can result in extensive DNA damage, processes for DNA protection and DNA repair are vital [40]. The microarray analysis revealed a significant increase in the transcription of *recA *(BCAL0953) in response to a 5 min treatment with NaOCl and a 30 min treatment with H_2_O_2 _(Table 2). In the qPCR experiments, no significant increase in transcription of *recA *was observed in the biofilms treated with H_2_O_2 _for 15 min (Table 2). In contrast, in the 30 min treated biofilms, the transcription of this gene was significantly increased. We also observed a slight upregulation of *recA *following a 60 min treatment (1.4-fold upregulation) but this was not statistically significant (p = 0.076). Our hypothesis is that, although most of the H_2_O_2 _has disappeared after 15 min, the transcriptional response to DNA damage caused by H_2_O_2 _is delayed. Upregulation of the *recA *gene has previously already been described for planktonic *P. aeruginosa *cultures treated with oxidizing agents [16,17].

No increased transcription of *dps *(BCAL3297), encoding the non-specific DNA-binding protein Dps, was neither observed in the H_2_O_2_- nor in the NaOCl-treated biofilms; these data are similar to previous observations in *Burkholderia pseudomallei *[41]. In *E. coli*, the transcription of *dps *is regulated by OxyR in exponentially growing planktonic cultures, whereas in stationary phase cultures, *dps *is transcribed in an RpoS-dependent manner [42,43]. The high expression of *dps *in the untreated biofilms and the absence of an increased expression in the ROS-treated biofilms suggest that RpoS rather than OxyR regulates the transcription of *dps *in mature *B. cenocepacia *biofilms [41,44].

### Upregulation of the *iscRSUA-hscAB-fdx-iscX *gene cluster

Iron-sulfur cluster-containing proteins are highly sensitive to the cellular redox status and they are very vulnerable to oxidants such as H_2_O_2 _[39,45]. In order to restore their function, iron-sulfur clusters are either repaired or they can be assembled *de novo *[45]. In *E. coli*, apo-IscR will increase the transcription of the *isc *gene cluster, and together with OxyR it will also stimulate the production of oxidation-resistant Suf proteins [46]. As no homologues of the *E. coli suf *gene cluster are found within the *B. cepacia *complex, the transcriptional response in the H_2_O_2_-treated biofilms only involves the increased transcription of the *iscSUA-hscAB-fdx-iscX *gene cluster (BCAL2192-BCAL2198; Table 2&3). In addition, an increase in the number of transcripts for *iscR *(BCAL2199) was also observed (Table 2&3). The latter gene has been identified in a recent screening of 1500 mutants of *P. aeruginosa *PAO1, which also lacks the *suf *operon, as an essential gene in the resistance to H_2_O_2 _[47]. Our data suggest that the increased transcription of the *iscRSUA-hscAB-fdx-iscX *gene cluster is important to regain intact iron-sulfur clusters in the ROS-treated *B. cenocepacia *J2315 cells.

As a consequence of the damage by oxidants to the iron-sulfur clusters, [4Fe-4S]^2+ ^and [2Fe-2S]^2+ ^containing enzymes, including aconitase B (AcnB), are inactivated [9]. Although AcnB is the major aconitase during normal growth conditions, it loses all activity in response to strong oxidants [48]. Usually, this drop in aconitase activity is compensated by the increased production of aconitase A (AcnA), which is invulnerable to oxidative inactivation *in vivo *[39,49]. However, in the present study no increased expression of *acnA *(BCAM0961) was observed, while the number of mRNA transcripts of *acnB *(BCAL1833) increased both in the H_2_O_2_- and in the NaOCl-treated biofilms; the data obtained for the H_2_O_2_-treated biofilms were confirmed by qPCR (Table 2). This observation is puzzling, yet it may be the result of an increased stability of *acnB *mRNA by the accumulating apo-aconitases, as previously observed in *E. coli *[48].

### Identification of ncRNA

The custom made 4 × 44K *B. cenocepacia *J2315 microarray does not only contain probes for protein encoding genes, but also 1,520 probes for selected IGs. A significant upregulation was observed in 39 (2.6%) and 56 (3.7%) of these IGs after treatments with H_2_O_2 _or NaOCl, respectively, whereas the expression of 54 (3.6%) and 68 (4.5%) IGs was significantly downregulated in response to these treatments (Table 1). A closer inspection of these IGs revealed that many are located in close proximity of genes with a similar expression pattern, suggesting that co-transcription of the IGs occurs synchronously with their adjacent genes. However, certain IGs demonstrated markedly different expression patterns compared to their flanking genes, and the basal expression levels of several of these IGs were also high. Of the 39 IGs, which showed a significant upregulation after exposure to H_2_O_2_, 11 displayed an expression pattern different from their flanking genes. These IGs include seven previously shown to be either significantly up- or downregulated in *B. cenocepacia *J2315 cells grown in sputum [14]. Two IGs, IG1_2935724 and IG1_3008003 identified by Yoder-Himes et al. [50] to contain putative ncRNA, were also overexpressed. Finally, the transcription of two IGs, IG1_950309 (Bc1) and IG1_2242852, which have been identified previously to contain putative ncRNA in a computational search [51], was also increased in response to exogenous oxidative stress.

**Figure 6:**
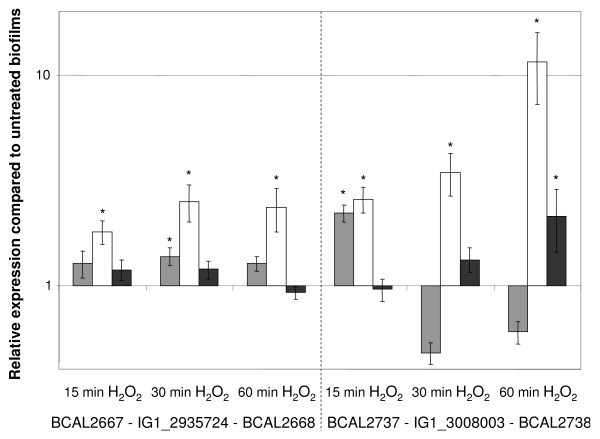
**Effect of treatments with H**_**2**_**O**_**2 **_**on the expression of the IGs IG1_2935724 and IG1_3008003 and their adjacent genes**. The expression of the genes BCAL2667 and BCAL2737 (grey bars) and BCAL2668 and BCAL2738 (black bars) and of the IGs (IG1_2935724 and IG1_3008003 [white bars]) in the treated biofilms is compared to the expression observed in the untreated biofilms. Error bars represent SEM. *: significant upregulation in treated biofilms compared to untreated biofilms (p < 0.05).

**Figure 7:**
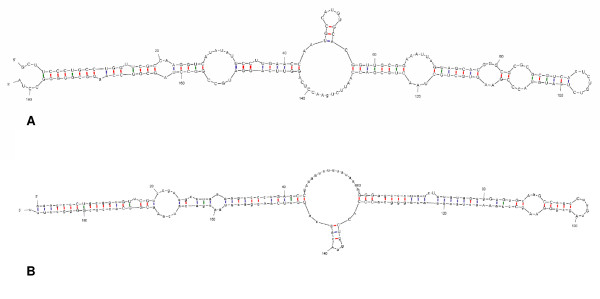
**Secondary structure of the putative 6S RNA in IG1_2935724 and the 6S RNA consensus structure**. A. Secondary structure obtained for the intergenic region spanning nucleotides 2935805 to 2935985 (*B. cenocepacia *J2315; chromosome 1) as predicted by mfold using standard settings [76]. B. Secondary structure of the 6S RNA consensus structure (RFAM database [53]).

Of the 56 IGs significantly upregulated in response to a NaOCl-treatment, 20 displayed a different expression pattern than their flanking genes. Again, a significant upregulation of the expression of IG1_2935724 and IG1_3008003 as well as IG1_950309 was observed. In order to confirm these microarray data, we performed qPCR experiments for IG1_2935724 and IG1_3008003 as well as for their flanking genes. The expression of IG1_2935724 was clearly higher when the biofilms were treated with H_2_O_2_, whereas no such upregulation was observed either for BCAL2667 (encoding a cell division protein ZapA) or for BCAL2668 (encoding a hypothetical protein) (Figure 6). In addition, the transcription of BCAL2667 and BCAL2668 in the untreated biofilms was clearly lower compared to the transcription of IG1_2935724 (data not shown). The secondary structure for the mRNA transcript (spanning the region from nucleotide 2935805 to nucleotide 2935985) has a marked similarity to the 6S RNA consensus structure (Figure 7) [52].

The qPCR results also revealed a significant upregulation of IG1_3008003 in the H_2_O_2_-treated biofilms (Figure 6). However, the transcription of the neighbouring genes BCAL2737 (coding for a pseudouridine synthase) and BCAL2738 (coding for a hypothetical protein) was not altered to the same extent and the C_T _values obtained for IG1_3008003 in the untreated biofilms were markedly lower than the corresponding C_T _values of BCAL2737 and BCAL2738. Although the presence of putative ncRNA in IG1_3008003 has been reported previously [50], no ncRNA was identified in this IG using the RFAM database [53].

Three IGs (IG2_551370; IG2_2731412 and IG2_794541) in which putative ncRNAs were identified in a computational search [51] were downregulated in the H_2_O_2_- as well as in the NaOCl-treated biofilms.

### Sigma factors

As the present study focussed on the changes in gene expression in sessile cells following treatment with high concentrations of ROS, major (general) stress responses were observed and the expression of multiple σ factors was increased (Table 2). These σ factors included an RNA polymerase σ^32 ^factor (BCAL0787), an ECF (extracytoplasmic function) σ^70 ^factor OrbS (BCAL1688), a putative σ factor (BCAL3478) and an ECF σ^70 ^factor FecI (BCAL1369).

The ECF σ^70 ^factor encoded by BCAL1688 has been annotated as OrbS or EcfI, a regulatory σ factor located adjacent to the ornibactin gene cluster in *B. cenocepacia *[54-56]. The transcription of *orbS *is regulated by the Fur repressor, which represses the *orbS *promoter when cells are growing under iron-replete conditions [56]. In correspondence to the high increase of the transcription of *orbS*, two of the genes belonging to the ornibactin gene cluster, *orbH *(BCAL1689) and *orbG *(BCAL1690), were also found to be upregulated (Table 3). The IG sequence between *orbS *and *orbH *also showed elevated expression (Table 3). However, none of the other genes present in the ornibactin gene cluster (*orbE *and *orbI-orbL*) were upregulated. Other studies have shown that *orbH *and *orbG *are cotranscribed with *orbS *[56], correlating to the increases we observed in these two genes and the intervening IG region. The lack of transcription of the other *orb *genes suggests that the levels of OrbS under our experimental conditions were insufficient to induce the transcription of *orbE *and *orbI-orbL *[57,58]. Alternatively, the transcription of *orbE *and *orbI-orbL *may depend on a post-transcriptional regulation of OrbS activity [59]. This posttranscriptional regulation might result from the interaction of OrbS with 6S RNA [52]. An increased transcription of a second σ^70 ^factor involved in iron-uptake, FecI (encoded by BCAL1369), was also observed. Similar observations have been described for H_2_O_2_-treated *P. aeruginosa *cells [16]. The increased transcription of *orbS *as well as of *fecI *suggests that the cells apparently suffer from some degree of iron starvation or that the Fur repressor function is transiently lost [16].

The exact roles of the σ factors, encoded by BCAL0787 and BCAL3478, in relation to these conditions of oxidative stress remain to be elucidated. Remarkably, the expression pattern of BCAL3478 is highly similar to that of its divergently transcribed neighbouring gene BCAL3477 (Table 3). Neither *rpoN *(σ^54^; BCAL0813), nor *rpoE *(σ^E^; BCAL0998 and BCAL2872) showed an increased transcription following exposure to exogenous ROS, corroborating previous data that neither RpoN nor RpoE are involved in the resistance towards H_2_O_2 _[60,61].

### Genes involved in motility and chemotaxis

*B. cepacia *complex bacteria are motile rods, equipped with flagella. These flagella are multi-component structures, which are synthesized and assembled in a complex process involving over 40 hierarchically expressed genes [62]. Flagella are not only important in the formation of biofilms, they also play a key role in the adherence to and the invasion into host cells [63]. In addition, intact flagella evoke host immune responses [64]. The data obtained in the microarray analysis reveal that many *B. cenocepacia *J2315 flagellum-related genes and genes involved in chemotaxis show an increased expression in biofilms exposed to exogenous ROS. The overexpressed genes in the H_2_O_2_-treated biofilms include those that belong to two major gene clusters: (i) BCAL0520-BCAL0525 and BCAL0526-BCAL0528, and (ii) BCAL0564-BCAL0572, BCAL0575 and BCAL0576-BCAL0577. The expression of the majority of these genes was also significantly increased in response to a short exposure to NaOCl (data not shown). In addition, the increased transcription of the flagellar motor protein encoding gene *motA *was also observed (the upregulation of *motB *was not significant). Furthermore, the transcription of five and nine genes coding for (putative) methyl-accepting chemotaxis proteins was increased in the H_2_O_2_- and NaOCl-treated biofilms, respectively. Although our data clearly indicate that sessile *B. cenocepacia *J2315 cells exposed to exogenous oxidative stress overexpress many flagellar and chemotaxis genes, previous experiments demonstrated that treatments with 0.3% H_2_O_2 _did not result in significant decreases in total biofilm biomass [7]. The latter observation suggests that the overexpression of these genes does not result in a global dispersal event. Further research is required to elucidate the role of the increased transcription of these genes in response to exogenous ROS.

### Phage-related genes

**Figure 8:**
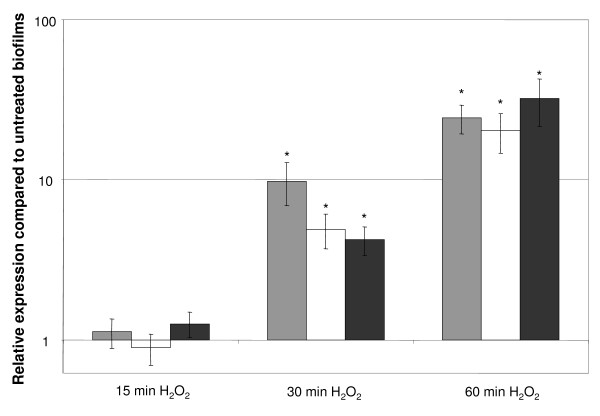
**Effect of treatments with H**_**2**_**O**_**2 **_**on the expression of three selected BcepMu prophage genes**. The expression of BCAS0543 (BcepMu12; grey bars), BCAS0546 (BcepMu9; white bars) and BCAS0547 (BcepMu8; black bars) in the H_2_O_2_-treated biofilms is compared with the expression observed in the untreated biofilms. Error bars represent SEM. *: significant upregulation in treated biofilms compared to untreated biofilms (p < 0.05).

The microarray analysis revealed a significant upregulation of multiple phage-related genes in sessile cells after exposure to H_2_O_2 _(Table 4). BcepMu is a Mu-like prophage that is present in many *B. cenocepacia *ET12 strains and it has been annotated BcenGI14 (*B. cenocepacia *J2315 Genomic Island 14; [13]). The prophage sequence was found to encode 53 proteins and it has been divided in three functional clusters, including a module involved in replication, regulation and pathogenesis [65]. The transcription of these genes probably results in a monocistronic mRNA as a similar upregulation of multiple IGs within this cluster was observed (Table 4). This prophage module contains many genes coding for small hypothetical proteins. In addition, it contains two genes (BcepMu8 and BcepMu9) that show high similarities to *Pseudomonas *phage B3 transposase B and A subunits (57% and 60% identity, respectively) [66,67]. The results of the qPCR analysis for three selected genes (BCAS0543 [BcepMu12], BCAS0546 [BcepMu9] and BCAS0547 [BcepMu8]) indicated that none of these genes was upregulated after a 15 min treatment with H_2_O_2_; yet, a significant upregulation was observed when *B. cenocepacia *J2315 biofilms were treated for 30 or 60 min (Figure 8). Thus far, the transcriptional mechanism resulting in the upregulation of these BcepMu prophage genes has not been elucidated.

**Table 4:** Genes of the BcepMu prophage that have an increased expression in H_2_O_2 _exposed *B. cenocepacia *J2315 biofilms (30 min).

Gene	BcepMu gene assignments	Annotation	**Fold change in expression in H**_**2**_**O**_**2**_**-treated biofilms (30 min) vs untreated biofilms**
IG3_584892		IG upstream of BCAS0540	8.5
BCAS0540	BcepMu16	Hypothetical protein	10.0
BCAS0540A	BcepMu15	Hypothetical protein	9.3
BCAS0540B	BcepMu14	Hypothetical protein	No probe present on array
BCAS0541	No BcepMu assignment	Hypothetical protein	9.6
IG3_601361		IG between BcepMu14 and BcepMu13	12.8
BCAS0542	BcepMu13	Hypothetical protein	7.7
BCAS0543	BcepMu12	Putative phage transcriptional regulator	10.1
BCAS0544	BcepMu11	Hypothetical protein	8.3
BCAS0545	BcepMu10	Hypothetical protein	1.7
BCAS0546	BcepMu9	Tn552/IS1604 rve transposase	2.6
BCAS0547	BcepMu8	Putative DNA-binding phage protein	3.8
BCAS0548	BcepMu7	Hypothetical protein	3.9
BCAS0549	BcepMu6	Hypothetical protein	3.7
IG3_607288		IG between BcepMu5 and BcepMu6	4.0
BCAS0550	BcepMu5	Hypothetical protein	3.7
BCAS0551	BcepMu4	Hypothetical protein	No probe present on array
IG3_608364		IG between BcepMu3 and BcepMu4	2.7
BCAS0552	BcepMu3	Single-stranded DNA binding protein	2.1
BCAS0553	BcepMu2	Mu gp16 gemA	1.7
BCAS0554	BcepMu1	Mu protein C/Mor gp17 transcription regulator	1.9

The results represent the fold change in expression in the treated biofilms compared to the expression in the untreated biofilms (based on the microarray analysis).

### Downregulated genes in the H_2_O_2_- and NaOCl-treated biofilms

Although 185 (2.6%) genes showed a significant downregulation of the transcription in biofilms exposed to H_2_O_2, _a more than five fold change in expression was only observed for 17 genes. Similarly, for 43 of the 331 (4.6%) downregulated genes in the NaOCl-treated biofilms, transcription was decreased more than five fold. A total of 117 (1.6%) genes was downregulated in response to both treatments. Despite the large numbers of genes showing a decreased expression in response to these ROS, no obvious patterns were observed. qPCR experiments were performed for two selected genes: BCAM1335 (encoding a glycosyltransferase) and BCAM1554 (encoding a diguanylate cyclase) and they confirmed that both genes were significantly downregulated in response to exposure to H_2_O_2 _(data not shown).

### Fold change correlation between qPCR experiments and microarray analysis

In order to determine the correlation between the fold changes in gene expression observed in the microarray analysis and the qPCR experiments, a regression analysis was performed. The results of the regression analysis between the log_2 _fold changes obtained in the qPCR experiments and the microarray analysis showed a good linear correlation between both datasets, with a correlation coefficient of 0.89 (p < 0.01) and a slope of 0.67. This suggests that the custom made 4 × 44K microarray for *B. cenocepacia *is highly reliable to evaluate changes in global gene expression in *B. cenocepacia *J2315.

## Conclusions

Our data show that the successful defence of sessile *B. cenocepacia *J2315 cells against high doses of ROS involves a plethora of protective mechanisms. These include mechanisms that allow cells to neutralize and scavenge ROS and mechanisms that are involved in the repair of the cellular damage resulting from the exposure to ROS. Several results in the present study corroborate data from previous studies that focussed on the transcriptional response to sublethal doses of exogenous ROS [15-21]. Rather unexpected observations included the high upregulation of three Ohr encoding genes in H_2_O_2_-treated sessile *B. cenocepacia *J2315 cells and the increased extracellular lipase activity in several H_2_O_2_-treated *B. cepacia *complex biofilms. In addition, our data revealed an increased transcription of multiple phage-related genes, IGs and chemotaxis- and motility-related genes in the treated biofilms. These observations contribute to a better insight in the molecular mechanisms involved in the resistance of sessile *B. cenocepacia *J2315 cells against exogenous ROS.

## Methods

### Strains and culture conditions

The strains used in the present study are shown in Table 5. All wild type strains were cultured routinely on Luria-Bertani agar (LBA, Oxoid, Hampshire, UK) at 37°C. Both *B. cenocepacia *MDL1 and MDL2 mutant strains were cultured on LBA containing trimethoprim (100 μg/ml; Certa, Braine-l'Alleud, Belgium).

**Table 5:** Strains used in the present study.

Strain	LMG number	Strain information	Reference
*B. cenocepacia *J2315	LMG 16656	ET12 strain, CF patient, UK	[77]
*B. cenocepacia *C5424	LMG 18827	Epidemic strain, CF patient, Canada	[78]
*B. cenocepacia *MDL1	-	C5424 *katA *mutant strain	[12]
*B. cenocepacia *MDL2	-	C5424 *katB *mutant strain	[12]
*B. multivorans *ATCC 17616	LMG 17588	Soil, USA	[79]
*B. cenocepacia *HI2424	LMG 24507	PHDC strain, soil, USA	[80]
*B. cenocepacia *AU1054	LMG 24506	PHDC strain, CF patient, USA	[81]
*B. ambifaria *AMMD	LMG 19182	Pea rhizosphere, USA	[82]
*B. dolosa *AU0158	LMG 24508	Epidemic strain, CF patient, USA	[83]

### Biofilm formation and treatment

Starting from an overnight culture, an inoculum suspension containing appr. 5 × 10^7 ^CFU/mL was prepared in Luria-Bertani broth (LBB). Subsequently, 100 μL of this suspension was added to the wells of a round-bottomed polystyrene 96-well microtiter plate (TPP, Trasadingen, Switzerland). After 4 h of adhesion at 37°C, the supernatant (containing non-adhered cells) was removed and the wells were rinsed using 100 μL saline (0.9% NaCl), 100 μL of sterile LBB was added to all wells and the biofilms were allowed to grow for an additional 20 h period (under static conditions). Preliminary experiments in which the number of cells present in these biofilms was quantified (by plating) indicated that 5 × 10^7 ^to 7 × 10^7 ^CFUs are present in each of these biofilms following 4 h of adhesion and 20 h of biofilm formation. The supernatant was then again removed and 120 μl of a 0.3% H_2_O_2 _solution (Acros Organics, Geel, Belgium) or 120 μl of a 0.02% NaOCl solution (Forever, Courcelles, Belgium) was added. H_2_O_2_-treated biofilms were exposed to this disinfectant for 15, 30 or 60 min; NaOCl-treated biofilms were exposed for 5 min. Similarly, 120 μL of saline was added to biofilms that served as untreated controls. After the prescribed contact times, the disinfectant/saline was removed. All disinfectant solutions were prepared using MQ water (Millipore, Billerica, MA, USA) and were filter-sterilized before use (Puradisk FP30; Whatman, Middlesex, UK).

### Resazurin-based viability staining

For all strains included in the present study, the fraction of surviving sessile cells in the H_2_O_2_-treated biofilms (15, 30 or 60 min) was determined by using a resazurin-based viability staining assay, as described previously [31]. In brief, after the various treatments, the supernatant was removed and 100 μl of sterile saline and 20 μl of CellTiter-Blue (Promega, Madison, WI, USA) was added to all wells. Plates were incubated aerobically for 1 h at 37°C and fluorescence (λ_exc_: 560 nm, λ_em_: 590 nm) was measured using a multilabel microtiter plate reader (Wallac Victor^2^, Perkin Elmer LAS, Waltham, MA, USA).

### RNA extraction, labelling and hybridization

In the microarray and qPCR experiments, treated and untreated *B. cenocepacia *J2315 cells were harvested by scraping and sonication (30 s; Branson 3510, Branson Ultrasonics Corp, Danbury, CT, USA) and transferred to sterile tubes. RNA was extracted using the Ambion RiboPure Bacteria Kit (Ambion, Austin, TX, USA) according to the manufacturer's instructions and the procedure included a DNase I treatment of 1 h at 37°C. After extraction, the RNA was concentrated using Microcon YM-50 filter devices (Millipore). Prior to the cDNA synthesis, RNA yields and RNA quality were assessed using the Agilent 2100 Bioanalyzer (Agilent, Santa Clara, CA, USA). The results of this control revealed that despite the harsh treatments imposed on the sessile cells, the extracted RNA fulfilled the stringent requirements for microarray experiments (data not shown). Subsequently, 10 μg of total RNA was used to synthesize labelled cDNA using a two-step amino-allyl procedure (CyScribe Post-Labelling Kit, GE Healthcare, Buckinghamshire, UK). Spike-in controls were included in the labelling procedure for quality control purposes (Agilent). The labelled cDNA was purified using the CyScribe GFX Purification Kit (GE Healthcare) and the fluorescent signal of each labelled sample was determined using electrophoretic separation of 1 μL of the final labelled cDNA in agarose, followed by scanning (GeneTAC GLS IV Scanner, Genomic Solutions Inc, Ann Arbor, MI, USA).

The hybridization and washing of the custom made 4 × 44K arrays was performed according to the manufacturer's instructions with some minor modifications. These modifications included the heat-denaturation of the mix of cDNA and blocking agent for 3 min at 98°C, followed by a cooling down to room temperature before addition of the hybridization buffer. After 17 h of hybridization at 65°C, the microarrays were washed and the washing routine included the use of an acetonitrile solution and the Agilent Stabilization and Drying Solution. The signal intensities were scanned using a microarray scanner (G2565 BA, Agilent) and processed using the Feature Extraction software (version 9.5.1., Agilent) with default settings. Three independent experiments were performed for treatments with 0.3% H_2_O_2 _(30 min) and 0.02% NaOCl (5 min). Similarly, six biological replicates were included for the untreated biofilms.

### Microarray experimental design and analysis

The custom-made 4 × 44K microarray for *B. cenocepacia *was previously developed using Agilent's two-colour 60-mer ink jet synthesis platform [68]. In the present study, a reference design was applied; to this end all Cy5 labelled samples were compared to a Cy3 labelled reference sample, which was generated by pooling RNA extracted in ten independent (untreated) biofilm experiments. The gene expression analysis was performed using GeneSpring GX 7.3 (Agilent) and data were normalized using the Affymetrix FE data normalization procedure recommended for two-colour Agilent microarrays. Only features dedicated to *B. cenocepacia *J2315 that were labelled "present" or "marginal" were included in the analysis. After these initial filter steps, an arbitrary cut-off value of a two-fold ratio change was applied to identify differentially expressed genes. Subsequently, a one way ANOVA analysis was performed (p < 0.05). The experimental protocols and the raw microarray data can be found in ArrayExpress under the accession number E-MEXP-2502.

### Quantitative RT-PCR

In order to validate the microarray results, the expression of 36 genes and two IGs in the H_2_O_2_-treated biofilms was examined by using RT-qPCR (Additional file 5). In addition to the confirmation of the microarray results for the 30 min treated biofilms, the expression of the selected genes was also examined in biofilms treated with H_2_O_2 _for 15 and 60 min. Five biological replicates were included for each test condition. Biofilm formation, treatments, RNA extraction and DNase I treatments were performed as described above. cDNA was synthesized using the SuperScript VILO cDNA Synthesis Kit (Invitrogen, Carlsbad, CA, USA) using 1 μg of total RNA as starting material. Subsequently, cDNA was diluted (1:10) in DEPC water (Invitrogen) and stored at -20°C until further use. Forward and reverse primers were developed using Primer Express Software (Applied Biosystems, Carlsbad, CA, USA) according to the manufacturer's instructions and they were compared to the *B. cenocepacia *J2315 database using BLAST to determine their specificity.

All qPCR experiments were performed on a Bio-Rad CFX96 Real-Time System C1000 Thermal Cycler. 2 μL FW and RV primers (final concentration 600 nM), 2 μl of cDNA sample, 4 μl of DEPC water and 10 μl of iQ SYBR Green Supermix (Bio-Rad, Hercules, CA, USA) were added to the wells of a 96-well hard shell PCR-plate (100923; Bio-Rad). Each sample was spotted in duplo and an interrun calibrator as well as control samples without added cDNA were included in each experiment. The initial 3 min denaturation step at 95°C was followed by 40 amplification cycles, consisting of 15 s at 95°C and 60 s at 58°C. A melting curve analysis was included at the end of each run.

In addition to the 36 genes described in Additional file 5, primers were also designed for ten candidate reference genes (Additional file 6). These ten genes included (i) five genes (BCAL1459, BCAL1659, BCAL2694, BCAM2784 and BCAS0175), which encode functionally diverse proteins and which showed a moderate and stable expression under all test conditions in the microarray experiments (fold difference between test conditions was between 0.9 and 1.1 and raw fluorescence values >300) and (ii) five of the housekeeping genes used in the *B. cepacia *complex MLST scheme (BCAL0036, BCAL0289, BCAL0421, BCAL1861 and BCAM0991) [69]. The gene stability analysis using GeNorm [70] revealed that all these candidate reference genes were stably expressed under all test conditions. Pairwise variation analysis (data not shown) resulted in the selection of five genes (BCAL1659, BCAL2694, BCAL1459, BCAS0175 and BCAL1861) necessary for an accurate normalization of our data.

### Lipase activity in biofilm supernatant

In order to determine the lipase activity in the supernatant of untreated and H_2_O_2_-treated biofilms, *B. cenocepacia *J2315, AU1054 and HI2424, *B. multivorans *LMG 17588, *B. ambifaria *LMG 19182 and *B. dolosa *AU0158 biofilms were grown as described above. Subsequently, 120 μl of 0.3% H_2_O_2 _(1% for *B. dolosa *AU0158) was added to the biofilms for 15, 30 or 60 min; 120 μl of saline was added to untreated control biofilms. After the prescribed contact times, the supernatants were collected separately, they were filter sterilized (0.2 μm) and retained on ice. Extracellular lipase activity was determined as described previously [38]. In brief, 20 μl of fluorogenic 4-MU based substrates (4-MU palmitate, 4-MU stearate or 4-MU oleate) was added to 180 μl sterile supernatant in black 96-well microtiter plates (CulturPlate-96F, Perkin Elmer, Waltham, MA, USA). Plates were incubated at 37°C and every 30 min, fluorescence was measured using a multilabel microtiter plate reader (Wallac Victor^2^) (λ_exc_: 355 nm, λ_em_: 460 nm). Three replicates were included per test condition and per fluorogenic substrate.

### Quantification of H_2_O_2 _in biofilm supernatant

To determine the importance of KatA and KatB for the survival of *B. cenocepacia *biofilms in the presence of high concentrations of H_2_O_2_, the degradation of H_2_O_2 _was monitored for *B. cenocepacia *J2315, C5424, MDL1 (*katA *mutant) and MDL2 (*katB *mutant) biofilms. To this end, supernatants were removed from the biofilms after 15, 30 and 60 min of treatment and H_2_O_2 _concentrations were determined titrimetrically using KMnO_4_, as described previously [71].

### Evaluation of the fold change correlation between qPCR experiments and microarray analysis

For each gene and IG examined by qPCR, the fold change in expression between the untreated and H_2_O_2_-treated (0.3%, 30 min) biofilms was compared to the corresponding fold change determined in the microarray analysis. To this end, the log_2 _fold changes were subjected to bivariate regression analysis and Pearson correlation coefficients were determined using SPSS 15 [72,73].

## Authors' contributions

EP performed all procedures associated with the biofilm experiments, the RNA extractions and the qPCR experiments, did all data analysis and wrote the first draft of the manuscript. AS performed the RNA quality control and all microarray experiments. EM provided assistance with the design of the microarray study. TC, EP and HN planned the original study and wrote the manuscript. All authors have read the final paper and contributed to its written content.

## Supplementary Material

Additional file 1 **Upregulated genes, intergenic regions and tRNA- and rRNA- encoding sequences in H**_**2**_**O**_**2**_**-treated biofilms**. Complete list of all *B. cenocepacia *J2315 genes, intergenic regions and tRNA- and rRNA- encoding sequences showing a significantly increased expression (>2-fold change; p < 0.05) in H_2_O_2_-treated biofilms compared to the expression in the untreated biofilms. Click here for file

Additional file 2 **Downregulated genes, intergenic regions and tRNA- and rRNA- encoding sequences in H**_**2**_**O**_**2**_**-treated biofilms**. Complete list of all *B. cenocepacia *J2315 genes, intergenic regions and tRNA- and rRNA- encoding sequences showing a significantly decreased expression (>2-fold change; p < 0.05) in H_2_O_2_-treated biofilms compared to the expression in the untreated biofilms. Click here for file

Additional file 3 **Upregulated genes, intergenic regions and tRNA- and rRNA- encoding sequences in NaOCl-treated biofilms**. Complete list of all *B. cenocepacia *J2315 genes, intergenic regions and tRNA- and rRNA- encoding sequences showing a significantly increased expression (>2-fold change; p < 0.05) in NaOCl-treated biofilms compared to the expression in the untreated biofilms. Click here for file

Additional file 4 **Downregulated genes, intergenic regions and tRNA- and rRNA- encoding sequences in NaOCl-treated biofilms**. Complete list of all *B. cenocepacia *J2315 genes, intergenic regions and tRNA- and rRNA- encoding sequences showing a significantly decreased expression (>2-fold change; p < 0.05) in NaOCl-treated biofilms compared to the expression in the untreated biofilms. Click here for file

Additional file 5 **FW and RV primers used in the qPCR experiments**. Click here for file

Additional file 6 **FW and RV primers used in the qPCR experiments for ten selected reference genes**. Five genes encoding functionally diverse proteins were selected based on their moderate and stable expression under all test conditions in the microarray experiments. The other selected candidate reference genes are five of the housekeeping genes used in the *B. cepacia *complex MLST scheme (in italic) [69]. Based on the pairwise variation analysis (GeNorm) [70], five genes (in bold) were selected for the normalization of the qPCR data. Click here for file
