# Supplementary material for: Transcriptional response of Burkholderia cenocepacia J2315 sessile cells to treatments with high doses of hydrogen peroxide and sodium hypochlorite
Source: BMC Genomics. 2010 Feb 5;11:90. doi: 10.1186/1471-2164-11-90 (PMC2830190; doi:10.1186/1471-2164-11-90)
Supplement: Additional file 1 — Upregulated genes, intergenic regions and tRNA- and rRNA- encoding sequences in H2O2-treated biofilms. Complete list of all B. cenocepacia J2315 genes, intergenic regions and tRNA- and rRNA- encoding sequences showing a significantly increased expression (>2-fold change; p < 0.05) in H2O2-treated biofilms compared to the expression in the untreated biofilms. [file 1471-2164-11-90-S1.PDF]

Gene name	Fold change	Annotation
BCAL0031	2.88	ATP synthase C chain
BCAL0032	2.49	ATP synthase B chain
BCAL0034	2.44	ATP synthase alpha chain
BCAL0036	2.31	ATP synthase beta chain
BCAL0037	2.04	ATP synthase epsilon chain
BCAL0042	7.48	bifunctional PutA protein
BCAL0110	2.33	putative aminotransferase
BCAL0114	3.18	flagellin (type II)
BCAL0126	3.45	chemotaxis protein MotA
BCAL0142	3.08	putative flagellar biosynthesis protein
BCAL0145	2.78	Adenosylhomocysteinase
BCAL0154	2.16	histone-like nucleoid-structuring (H-NS)
BCAL0155	2.05	putative cation efflux protein
BCAL0193	3.79	putative exported protein
BCAL0212	6.17	putative phenylacetic acid degradation NADH
BCAL0215	5.40	phenylacetic acid degradation protein PaaB
BCAL0261	2.25	50S ribosomal protein L17
BCAL0264	2.10	delta-aminolevulinic acid dehydratase
BCAL0282	2.35	putative ABC transporter extracellular solute-
BCAL0305	2.12	putative exported protein
BCAL0340	2.46	putative lipoprotein
BCAL0408	5.05	putative phenylacetic acid degradation
BCAL0455	2.83	putative hydrolase
BCAL0468	5.26	metallo peptidase, subfamily M48B
BCAL0524	2.12	flagellar motor switch protein FliG
BCAL0525	2.19	flagellar M-ring protein FliF
BCAL0527	4.49	flagellar protein FliS
BCAL0528	2.93	conserved hypothetical protein
BCAL0537	2.08	endonuclease/exonuclease/phosphatase family
BCAL0564	2.59	flagellar basal-body rod protein FlgB (putative
BCAL0566	3.20	basal-body rod modification protein FlgD
BCAL0567	4.17	flagellar hook protein 1 FlgE1
BCAL0568	3.31	flagellar basal-body rod protein FlgF (putative
BCAL0569	3.16	flagellar basal-body rod protein FlgG (distal
BCAL0571	3.01	flagellar P-ring protein precursor (basal body
BCAL0575	3.07	YcgR family protein
BCAL0576	4.31	flagellar hook-associated protein 1 (HAP1)
BCAL0577	4.49	flagellar hook-associated protein 3 (HAP3)
BCAL0580	8.08	putative chromate transport protein
BCAL0600	2.77	putative glutamine synthetase
BCAL0677	2.83	thiol:disulfide interchange protein
BCAL0683	2.14	conserved hypothetical protein
BCAL0708	2.47	conserved hypothetical protein

Gene name	Fold change	Annotation
BCAL0722	2.46	C4-dicarboxylate transport protein
BCAL0762	2.79	putative methyl-accepting chemotaxis protein
BCAL0763	3.58	putative exported protein
BCAL0771	37.71	non-heme chloroperoxidase
BCAL0783	3.69	putative membrane protein
BCAL0784	3.11	cytochrome d ubiquinol oxidase subunit II
BCAL0785	4.92	cytochrome d ubiquinol oxidase subunit I
BCAL0786	3.42	putative membrane protein
BCAL0787	4.67	RNA polymerase σ^{32}-factor
BCAL0831	3.87	putative storage protein
BCAL0833	4.11	putative Acetoacetyl-CoA reductase
BCAL0899	2.65	lactoylglutathione lyase
BCAL0940	6.51	putative transglycosylase
BCAL0943	2.81	conserved hypothetical protein
BCAL0953	2.58	putative recombinase A
BCAL0977	2.00	acetyltransferase (GNAT) family protein
BCAL1033	2.19	putative exported protein
BCAL1051	2.02	radical SAM superfamily protein
BCAL1071	3.22	NAD dependent epimerase/dehydratase family
BCAL1092	2.26	ABC transporter extracellular solute-binding
BCAL1103	2.00	putative OsmB-like lipoprotein
BCAL1105	6.81	putative exported protein
BCAL1106	4.07	cytochrome b561 family protein
BCAL1107	2.67	putative oxidoreductase
BCAL1146	2.38	AraC family regulatory protein
BCAL1236	4.61	putative exported protein
BCAL1368	2.32	putative porin
BCAL1369	2.22	putative RNA polymerase σ-factor FecI
BCAL1475	2.26	putative polysaccharide deacetylase
BCAL1610	2.28	periplasmic cystine-binding protein
BCAL1635a	3.85	putative exported protein
BCAL1662	2.62	putative methyl-accepting chemotaxis protein
BCAL1674	3.17	multidrug efflux system AmrA protein
BCAL1677	2.69	putative type-1 fimbrial protein
BCAL1688	6.45	putative RNA polymerase σ-factor
BCAL1689	3.83	MbtH-like protein
BCAL1690	2.26	putative dioxygenase
BCAL1752	3.29	conserved hypothetical protein
BCAL1761	2.40	MarR family regulatory protein
BCAL1763	5.48	putative exported protein
BCAL1764	5.25	putative exported protein
BCAL1765	7.51	putative exported protein
BCAL1766	18.02	OsmC-like protein
BCAL1824	3.09	putrescine-binding periplasmic protein
BCAL1829	6.13	putative outer membrane protein
BCAL1830	6.85	putative 2-nitropropane dioxygenase

Gene name	Fold change	Annotation
BCAL1837	2.09	conserved hypothetical protein
BCAL1888	2.03	putative membrane protein
BCAL1961	2.06	putative exported protein
BCAL1992	2.24	putative acyl-CoA thioesterase precursor
BCAL2013	2.11	AhpC/TSA family protein
BCAL2014	2.68	carboxymuconolactone decarboxylase family
BCAL2044	2.55	muramoyltetrapeptide carboxypeptidase
BCAL2123	2.28	conserved hypothetical protein
BCAL2144	2.56	ubiquinol oxidase polypeptide II precursor
BCAL2191	2.99	putative membrane protein
BCAL2192	2.42	conserved hypothetical protein
BCAL2193	2.46	ferredoxin, 2Fe-2S
BCAL2194	2.39	chaperone protein HscA homologue
BCAL2195	2.33	co-chaperone protein HscB homologue
BCAL2197	3.41	putative iron-sulfur cluster scaffold protein
BCAL2198	2.89	cysteine desulfurase
BCAL2199	2.29	putative transcriptional regulator protein
BCAL2201	2.21	putative membrane protein
BCAL2213	2.50	oligopeptidase A
BCAL2297	9.97	conserved hypothetical protein
BCAL2299	2.67	putative permease protein
BCAL2300	4.21	putative exported protein
BCAL2301	4.60	putative exported protein
BCAL2308	3.51	conserved hypothetical protein
BCAL2428	2.42	putative cytochrome C precursor-related protein
BCAL2429	2.18	putative cytochrome C precursor-related protein
BCAL2442	3.22	chaperone protein HtpG
BCAL2468	2.55	putative membrane protein
BCAL2500	2.17	hypothetical protein
BCAL2538	2.59	TetR family regulatory protein
BCAL2544	3.64	TetR family regulatory protein
BCAL2545	2.92	Major Facilitator Superfamily protein
BCAL2547	2.11	probe overlaps with the beginning of genen BCAL2547a (hypothetical protein)
BCAL2581_J_1	2.30	putative transposase-related protein
BCAL2584	2.93	metallo-beta-lactamase superfamily protein
BCAL2585	2.69	putative nitrilase
BCAL2586	2.62	AraC family regulatory protein
BCAL2605	2.04	two-component regulatory system, sensor kinase
BCAL2606	3.62	two-component regulatory system, response
BCAL2607	3.99	putative exported protein
BCAL2685	6.09	putative sulfite reductase
BCAL2703	3.30	conserved hypothetical protein
BCAL2735	2.51	isocitrate dehydrogenase [NADP]
BCAL2736	3.08	isocitrate dehydrogenase
BCAL2739	2.27	elongation factor G

Gene name	Fold change	Annotation
BCAL2752	2.04	putative membrane protein
BCAL2780	2.85	putative thioredoxin protein
BCAL2783	2.90	putative cyclopropane-fatty-acyl-phospholipid
BCAL2791	3.34	putative kynureninase
BCAL2792	3.59	putative tryptophan 2,3-dioxygenase
BCAL2899	2.63	4Fe-4S ferredoxin
BCAL2933	3.03	D-amino acid dehydrogenase small subunit
BCAL2934	2.15	electron transfer flavoprotein alpha-subunit
BCAL3003	3.18	hypothetical protein
BCAL3006	2.05	cold shock-like protein
BCAL3033	2.23	probable outer-membrane lipoproteins carrier
BCAL3035	2.79	thioredoxin reductase
BCAL3094	3.18	oxygen-independent coproporphyrinogen III
BCAL3146	2.30	60 kDa chaperonin 1
BCAL3147	3.46	10 kDa chaperonin 1
BCAL3227	3.10	conserved hypothetical protein
BCAL3231	2.24	hypothetical protein
BCAL3239	2.16	glucosyltransferase
BCAL3272	2.79	putative heat shock protein
BCAL3311	2.15	putative exported protein
BCAL3359	3.43	putative glutamate dehydrogenase
BCAL3362	3.71	putative oxidoreductase
BCAL3380	2.02	putative allantoicase
BCAL3420	2.01	biotin carboxyl carrier protein of acetyl-CoA
BCAL3472	2.38	conserved hypothetical protein
BCAL3473	3.48	putative outer membrane porin
BCAL3477	2.68	putative catalase
BCAL3478	3.41	putative RNA polymerase σ-factor
BCAL3479	2.32	putative transmembrane regulator
BCAL3490	2.39	putative exported protein
BCAL3492	2.12	putative exported protein
BCAL3505	2.85	flagellar motor switch protein FliN
BCAL3508	2.09	LrgB family protein
BCAL3514	3.18	outer membrane efflux protein
BCAM0007	2.32	putative phage integrase
BCAM0010	6.61	2-amino-3-ketobutyrate coenzyme A ligase
BCAM0011	5.21	threonine 3-dehydrogenase
BCAM0083	2.85	hypothetical protein
BCAM0165	7.72	conserved hypothetical protein
BCAM0166	3.40	NADH dehydrogenase
BCAM0276	2.53	putative universal stress protein
BCAM0277	2.04	conserved hypothetical protein
BCAM0278	7.26	putative heat shock protein
BCAM0280	6.87	putative phospholipid-binding protein
BCAM0280A	3.46	conserved hypothetical protein
BCAM0284	3.06	putative cytochrome c

Gene name	Fold change	Annotation
BCAM0285	2.81	conserved hypothetical protein
BCAM0290	3.57	putative universal stress protein
BCAM0291	3.64	putative universal stress protein
BCAM0292	2.89	putative universal stress protein
BCAM0293	2.58	putative acetate kinase
BCAM0294	5.34	putative universal stress protein
BCAM0295	4.87	conserved hypothetical protein
BCAM0296	3.32	acetoacetyl-CoA reductase
BCAM0297	3.31	putative polymerase
BCAM0298	2.49	putative phosphate acetyl/butyryl transferase
BCAM0299	3.76	putative zinc-binding alcoholdehydrogenase
BCAM0300	2.01	metallo-beta-lactamase superfamily protein
BCAM0306	2.66	putative membrane protein
BCAM0307	3.85	conserved hypothetical protein
BCAM0308	3.88	conserved hypothetical protein
BCAM0309	3.92	putative cell division-related metallo
BCAM0311	6.20	putative 6-phosphofructokinase
BCAM0312	2.58	putative polysaccharide deacetylase
BCAM0316	2.73	conserved hypothetical protein
BCAM0317	2.03	putative membrane protein
BCAM0405	6.23	putative trans-aconitate 2-methyltransferase
BCAM0414	2.74	conserved hypothetical protein
BCAM0548	2.67	60 kDa chaperonin 2
BCAM0661	3.42	CoA-transferase family III protein
BCAM0710	2.07	metallo peptidase, family M35
BCAM0740	22.05	conserved hypothetical protein
BCAM0741	2.52	LysR family regulatory protein
BCAM0772	2.61	putative membrane protein
BCAM0773	2.30	putative exported protein
BCAM0776	2.30	putative cNMP-binding domain protein
BCAM0778	2.03	OmpA family protein
BCAM0800	2.01	conserved hypothetical protein
BCAM0810	2.70	putative aromatic oxygenase
BCAM0851	2.76	conserved hypothetical protein
BCAM0896	36.48	putative organic hydroperoxide resistance
BCAM0906	2.13	putative dienelactone hydrolase family protein
BCAM0930	2.73	putative ankyrin-like protein
BCAM0931	5.68	catalase precursor
BCAM0987	3.76	flagellar hook protein 2 FlgE2
BCAM1012	2.41	putative histone-like protein
BCAM1015	3.16	putative porin
BCAM1111	4.18	ornithine decarboxylase
BCAM1216	15.54	alkyl hydroperoxide reductase subunit F
BCAM1217	41.26	alkyl hydroperoxide reductase subunit C
BCAM1219	2.39	putative ligand-binding receptor
BCAM1276	2.43	ArsR family regulatory protein

Gene name	Fold change	Annotation
BCAM1316a	2.74	conserved hypothetical protein
BCAM1316b	2.35	conserved hypothetical protein
BCAM1354	2.41	putative membrane protein
BCAM1364	2.12	putative NAD dependent epimerase/dehydratase
BCAM1374	2.41	conserved hypothetical protein
BCAM1420	2.43	efflux system transport protein
BCAM1421	2.28	RND family efflux system transporter protein
BCAM1433	8.78	putative short chain dehydrogenase
BCAM1500	2.15	putative universal stress protein
BCAM1502	4.23	conserved hypothetical protein
BCAM1503	4.09	putative methyl-accepting chemotaxis protein
BCAM1570	2.89	alcohol dehydrogenase
BCAM1572	2.96	methyl-accepting chemotaxis protein
BCAM1606	2.30	electron transfer flavoprotein, alpha subunit
BCAM1622	3.97	BNR/Asp-box repeat protein
BCAM1744	4.89	serine peptidase, family S9
BCAM1754	2.65	putative mechanosensitive ion channel
BCAM1765	2.89	putative oxalate decarboxylase
BCAM1777A	5.53	putative exported protein
BCAM1780	3.07	peptidoglycan-binding lysm:peptidase m23b
BCAM1787	2.67	putative porin
BCAM1804	8.23	methyl-accepting chemotaxis protein
BCAM1814	2.50	putative flavin-binding monooxygenase
BCAM1833	5.07	aconitate hydratase
BCAM1878	2.08	putative phage repressor protein
BCAM1911	2.11	hypothetical phage protein
BCAM1912	3.41	hypothetical phage protein
BCAM1963	4.45	conserved hypothetical protein
BCAM1966	2.50	ArsR family regulatory protein
BCAM1967	3.39	putative xenobiotic reductase
BCAM2006	6.44	putative aspartate carbomyltransferase
BCAM2039	2.34	putative transcriptional regulator
BCAM2076	2.30	putative diaminopimelate decarboxylase
BCAM2224	2.94	putative pyochelin receptor protein FptA
BCAM2231	4.25	transcriptional regulator PchR
BCAM2240	2.36	AraC family regulatory protein
BCAM2286	2.43	putative short chain dehydrogenase
BCAM2394	2.38	GntR family regulatory protein
BCAM2395	3.48	putative dehydrogenase/oxidoreductase protein
BCAM2396	3.04	conserved hypothetical protein
BCAM2415	2.04	putative NADPH-dependent FMN reductase
BCAM2484	2.47	5-methyltetrahydropteroyltriglutamate--homocysteine
BCAM2519	2.69	putative LysE family transporter
BCAM2529	2.23	ABC transporter ATP-binding protein
BCAM2536	2.18	putative alpha-beta hydrolase
BCAM2537	2.03	putative membrane protein

Gene name	Fold change	Annotation
BCAM2568	2.58	putative beta-ketoadipyl CoA thiolase
BCAM2569	5.02	IclR family regulatory protein
BCAM2584	4.39	putative gram-negative porin
BCAM2586	2.08	putative lactonase
BCAM2589	3.85	IclR family regulatory protein
BCAM2594	3.75	putative alcohol dehydrogenase
BCAM2595	2.25	putative carboxymuconolactone dehydrogenase
BCAM2609	2.26	putative exported protein
BCAM2620	3.79	putative lipoprotein
BCAM2621_J_0	2.35	putative porin-related protein (pseudogene)
BCAM2650	2.60	putative short-chain dehydrogenase/reductase
BCAM2677	5.76	putative membrane protein
BCAM2708	2.14	IclR family regulatory protein
BCAM2761	2.03	giant cable pilus
BCAM2837_J_0	2.32	two-component regulatory system, response
BCAM2837_J_1	2.97	two-component regulatory system, response
BCAS0081	2.01	ABC transporter ATP-binding membrane protein
BCAS0084	5.94	TetR family regulatory protein
BCAS0085	49.30	organic hydroperoxide resistance protein
BCAS0086	96.60	putative lipase
BCAS0151	2.53	hypothetical protein
BCAS0186	16.44	putative acyl carrier protein phosphodiesterase
BCAS0319	2.20	putative oxidoreductase
BCAS0320	2.17	isoquinoline 1-oxidoreductase alpha subunit
BCAS0540	9.99	hypothetical phage protein
BCAS0541	9.59	hypothetical phage protein
BCAS0542	7.70	hypothetical phage protein
BCAS0543	10.10	putative phage transcriptional regulator
BCAS0544	8.30	hypothetical phage protein
BCAS0546	2.61	putative phage integrase
BCAS0547	3.84	putative phage DNA-binding protein
BCAS0548	3.94	hypothetical phage protein
BCAS0549	3.68	hypothetical phage protein
BCAS0550	3.74	hypothetical phage protein
BCAS0552	2.09	hypothetical phage protein
BCAS0605	3.23	NmrA-like family protein
BCAS0637	3.45	60 kDa chaperonin 3
BCAS0638	5.60	10 kDa chaperonin 3
BCAS0640	3.45	conserved hypothetical protein
BCAS0681	2.05	putative transposase (pseudogene)
pBCA001	2.17	putative partition protein
pBCA002	2.24	putative partitioning protein
pBCA087	2.72	NUDIX hydrolase family protein
IG1_1014163	2.11	interG_chr1_pos_558_1014163:1014489
IG1_1507827	2.02	interG_chr1_pos_812_1507827:1507928

Gene name	Fold change	Annotation
IG1_1847123	2.87	interG_chr1_pos_1009_1847123:1847207
IG1_2221074	2.17	interG_chr1_pos_1150_2221074:2221262
IG1_2242852	2.27	interG_chr1_pos_1164_2242852:2244472
IG1_2420168	2.62	interG_chr1_pos_1223_2420168:2420460
IG1_2543049	2.27	interG_chr1_pos_1258_2543049:2543266
IG1_2843061	2.91	interG_chr1_pos_1403_2843061:2843803
IG1_2935724	6.44	interG_chr1_pos_1444_2935724:2936297
IG1_3008003	3.69	interG_chr1_pos_1476_3008003:3008659
IG1_3180283	2.53	interG_chr1_pos_1537_3180283:3180384
IG1_3295009	2.06	interG_chr1_pos_1607_3295009:3295231
IG1_3327989	2.16	interG_chr1_pos_1626_3327989:3328123
IG1_37663	2.51	interG_chr1_pos_28_37663:37730
IG1_3828501	2.59	interG_chr1_pos_1835_3828501:3828687
IG1_443956	2.25	interG_chr1_pos_277_443956:444522
IG1_516822	2.07	interG_chr1_pos_315_516822:516920
IG1_52439	2.01	interG_chr1_pos_35_52439:52522
IG1_619308	2.77	interG_chr1_pos_364_619308:619404
IG1_64305	3.38	interG_chr1_pos_43_64305:65908
IG1_687802	2.57	interG_chr1_pos_396_687802:688965
IG1_825611	3.32	interG_chr1_pos_472_825611:825836
IG1_901672	13.70	interG_chr1_pos_504_901672:902168
IG1_950309	2.59	interG_chr1_pos_528_950309:950472
IG2_1126199	2.21	interG_chr2_pos_582_1126199:1126466
IG2_1660910	2.27	interG_chr2_pos_846_1660910:1661218
IG2_2119934	2.10	interG_chr2_pos_1066_2119934:2120048
IG2_369070	2.16	interG_chr2_pos_168_369070:370084
IG2_429106	4.52	interG_chr2_pos_211_429106:432827
IG2_433416	2.37	interG_chr2_pos_212_433416:433537
IG3_584892	8.50	interG_chr3_pos_269_584892:600856
IG3_601100	9.34	interG_chr3_pos_270_601100:601231
IG3_601361	12.79	interG_chr3_pos_271_601361:601465
IG3_607288	3.97	interG_chr3_pos_279_607288:607409
IG3_608364	2.70	interG_chr3_pos_281_608364:608456
BCAL0181	2.62	intergenic region between BCAL0180 and BCAL0181
BCAL1136	2.69	intergenic region between BCAL1132 and BCAL1138
BCALr1899	4.13	Bacterial signal recognition particle RNA (RF00169)
BCALr3349	2.32	Bacterial RNase P class A (RF00010)
BCALr0080	4.89	tRNA Arg anticodon CCG, Cove score 82.41
BCALr0218c	2.67	tRNA Thr anticodon GGT, Cove score 90.12
BCALr0409c	2.58	tRNA Ala anticodon TGC, Cove score 90.89
BCALr0457	4.17	tRNA Lys anticodon CTT, Cove score 94.68
BCALr0472	3.46	tRNA Phe anticodon GAA, Cove score 86.97
BCALr0800	3.19	tRNA Gln anticodon TTG, Cove score 73.12
BCALr0949	5.06	tRNA Met anticodon CAT, Cove score 88.36
BCALr0970a	5.84	tRNA Asn anticodon GTT, Cove score 85.87

Gene name	Fold change	Annotation
BCALr1279	3.19	tRNA Pro anticodon TGG, Cove score 89.00
BCALr1290	2.13	tRNA Arg anticodon TCT, Cove score 88.96
BCALr1551a	5.11	tRNA Leu anticodon CAG, Cove score 72.78
BCALr1614	3.19	tRNA Met anticodon CAT, Cove score 86.01
BCALr2125f	4.07	tRNA Glu anticodon TTC, Cove score 60.10
BCALr2145	2.43	tRNA Ser anticodon GCT, Cove score 69.97
BCALr2219	3.08	tRNA Met anticodon CAT, Cove score 86.80
BCALr2236	4.79	tRNA Val anticodon CAC, Cove score 91.15
BCALr2281	3.05	tRNA Ser anticodon GGA, Cove score 70.94
BCALr3029	3.00	tRNA Ser anticodon CGA, Cove score 71.72
BCALr3075	2.93	tRNA Gly anticodon CCC, Cove score 61.13
BCAMr0918	2.83	tRNA Met anticodon CAT, Cove score 71.40
BCAMr2491b	3.41	23S RNA
BCAMr2491e	2.44	16S RNA
BCASr0743b	4.61	23S RNA
BCASr0743e	2.06	16S RNA
BCALr0409d	3.47	23S RNA
BCALr2231e	2.52	16S RNA
