# Supplementary material for: Transcriptional response of Burkholderia cenocepacia J2315 sessile cells to treatments with high doses of hydrogen peroxide and sodium hypochlorite
Source: BMC Genomics. 2010 Feb 5;11:90. doi: 10.1186/1471-2164-11-90 (PMC2830190; doi:10.1186/1471-2164-11-90)
Supplement: Additional file 2 — Downregulated genes, intergenic regions and tRNA- and rRNA- encoding sequences in H2O2-treated biofilms. Complete list of all B. cenocepacia J2315 genes, intergenic regions and tRNA- and rRNA- encoding sequences showing a significantly decreased expression (>2-fold change; p < 0.05) in H2O2-treated biofilms compared to the expression in the untreated biofilms. [file 1471-2164-11-90-S2.PDF]

Gene name	Fold change	Annotation
BCAL0001	2.12	sodium/hydrogen exchanger family protein
BCAL0368	3.10	cold shock-like protein CspD
BCAL0539	2.67	putative nitroreductase/p-nitrobenzoate
BCAL0625	4.46	LysR family regulatory protein
BCAL0665	3.86	dethiobiotin synthetase
BCAL0709	2.15	putative lipoate-protein ligase B
BCAL0759	2.20	UbiA prenyltransferase family protein
BCAL0861	2.15	Major Facilitator Superfamily protein
BCAL0961	2.91	putative lipoprotein
BCAL1161	2.38	conserved hypothetical protein
BCAL1176	2.11	putative fusaric acid resistance transport
BCAL1202	2.36	putative membrane protein
BCAL1203	2.38	conserved hypothetical protein
BCAL1380	3.06	(R)-stereoselective amidase
BCAL1520	2.16	putative lipoprotein
BCAL1549	3.27	putative sugar ABC transport system, membrane
BCAL1618	2.07	putative membrane protein
BCAL1631	2.16	conserved hypothetical protein
BCAL1776	2.11	AraC family regulatory protein
BCAL1844	2.39	conserved hypothetical protein
BCAL1936	2.04	AhpC/TSA family protein
BCAL2005	2.24	putative membrane protein
BCAL2006	2.21	hypothetical protein
BCAL2163	2.41	Cupin superfamily protein
BCAL2182	2.09	conserved hypothetical protein
BCAL2279	2.08	conserved hypothetical protein (pseudogene)
BCAL2354	2.04	2-isopropylmalate synthase
BCAL2358	2.24	acetolactate synthase isozyme III small subunit
BCAL2381	4.95	potassium-transporting ATPase C chain
BCAL2382	5.65	potassium-transporting ATPase B chain
BCAL2383	5.75	potassium-transporting ATPase A chain
BCAL2397	3.85	putative lipoprotein
BCAL2439	2.28	putative membrane protein
BCAL2450	3.19	putative metal transport integral membrane
BCAL2520	3.16	putative lipoprotein
BCAL2562	2.80	putative integrase (fragment)
BCAL2568	2.06	putative DNA repair protein (fragment)
BCAL2624	2.57	putative aromatic hydrocarbon catabolic
BCAL2625	2.87	Major Facilitator Superfamily protein
BCAL2648	2.15	putative outer membrane protein
BCAL2796	3.33	putative benzoylformate decarboxylase
BCAL2811	2.56	sorbitol dehydrogenase
BCAL2834	2.26	putative acylhydrolase
BCAL2843	2.58	putative branched-chain amino acid transport
BCAL2950	2.28	30S ribosomal protein S1

Gene name	Fold change	Annotation
BCAL3028	2.04	acetyltransferase (GNAT) family protein
BCAL3039	2.65	ABC transporter, membrane permease
BCAL3040	2.90	ABC transporter, membrane permease
BCAL3072	2.01	LysE type translocator
BCAL3183	2.72	putative hydrolase
BCAL3184	2.37	homogentisate 1,2-dioxygenase
BCAL3185	2.91	4-hydroxybenzoate transporter
BCAL3186	2.13	conserved hypothetical protein
BCAL3187	3.07	putative oxidoreductase
BCAL3237	2.14	putative transposase
BCAL3283	3.45	conserved hypothetical protein
BCAL3288	2.65	putative glycolate oxidase subunit GlcD
BCAL3317	2.51	putative membrane protein
BCAL3406	2.24	putative dehydrogenase
BCAL3409	2.23	IclR family regulatory protein
BCAM0013	8.00	putative acetyltransferase - GNAT family
BCAM0047	4.46	putative transporter - LysE family
BCAM0153	3.34	2-keto-3-deoxygluconate permease
BCAM0168	2.33	AraC family regulatory protein
BCAM0169	2.43	NmrA-like family protein
BCAM0203	2.20	putative membrane protein
BCAM0245_J_0	2.72	putative pyridoxal-dependent decarboxylase
BCAM0253	2.13	putative 3-oxoacyl-[acyl-carrier-protein]
BCAM0254	2.07	putative acyl carrier protein
BCAM0289	2.23	two-component regulatory system, sensor kinase
BCAM0432	2.86	putative exported protein
BCAM0457	2.33	hypothetical protein
BCAM0465	2.43	Major Facilitator Superfamily protein
BCAM0544	11.16	putative acetylglutamate kinase
BCAM0545	11.60	putative PTS transport system
BCAM0545a	5.10	putative membrane protein
BCAM0546	2.32	hypothetical protein
BCAM0547	2.72	putative LysE type translocator
BCAM0568	2.19	putative short chain dehydrogenase
BCAM0582	2.16	putative exported protein
BCAM0605	2.29	AnsC family regulatory protein
BCAM0705	4.84	putative membrane protein
BCAM0731	2.89	MarR family regulatory protein
BCAM0756	2.02	GntR family regulatory protein
BCAM0758	5.13	conserved hypothetical protein
BCAM0809	2.00	AraC family regulatory protein
BCAM0881	3.34	putative alpha amylase-family protein
BCAM0893	3.75	PAP2 superfamily protein
BCAM0993	2.02	tryptophan synthase alpha chain
BCAM1070	2.01	hypothetical phage protein
BCAM1071	2.80	hypothetical phage protein

Gene name	Fold change	Annotation
BCAM1080	2.28	hypothetical phage protein
BCAM1081	2.19	hypothetical phage protein
BCAM1100	2.81	LysR family regulatory protein
BCAM1102	2.07	Major Facilitator Superfamily protein
BCAM1126	2.05	putative exported protein
BCAM1149	2.16	putative lipoprotein
BCAM1152	4.35	Major Facilitator Superfamily protein
BCAM1163	2.04	conserved hypothetical protein
BCAM1249	2.01	putative CDP-alcohol phosphatidyltransferase
BCAM1335	3.65	glycosyltransferase
BCAM1410	2.95	putative lipoprotein
BCAM1466	3.83	IclR family regulatory protein
BCAM1467	2.15	periplasmic solute-binding protein
BCAM1476	2.14	AnsC family regulatory protein
BCAM1517	2.24	IclR family regulatory protein
BCAM1554	3.69	putative diguanylate cyclase
BCAM1573	2.03	alpha,alpha-trehalose-phosphate synthase
BCAM1602	2.08	conserved hypothetical protein
BCAM1619	3.02	putative DNA-binding cold-shock protein
BCAM1668	2.04	conserved hypothetical protein
BCAM1676	9.62	putative nitrite/sulfite reductase
BCAM1677	10.09	conserved hypothetical protein
BCAM1724	2.03	MarR family regulatory protein
BCAM1726	2.70	putative exported protein
BCAM1728	2.18	LysR family regulatory protein
BCAM1733	2.49	putative membrane protein
BCAM1771	2.23	putative ABC transporter, permease protein
BCAM1810	6.02	putative cold shock protein
BCAM1822	2.99	putative NAD-dependent glutamate dehydrogenase
BCAM1829	3.01	putative universal stress protein
BCAM1830	4.52	putative exported protein
BCAM1831	4.13	putative cyclase
BCAM1852	2.12	[2Fe-2S]-binding protein
BCAM1951	2.25	TetR family regulatory protein (pseudogene)
BCAM2011	2.81	conserved hypothetical protein
BCAM2035	2.08	putative exopolysaccharide biosynthesis protein
BCAM2064	2.20	putative periplasmic trehalase precursor
BCAM2081	3.15	conserved hypothetical protein
BCAM2112a	2.45	putative peptidase/dipeptidase (fragment)
BCAM2114	3.80	putative hydroxylase
BCAM2115	2.51	putative 4-hydroxyphenylacetate 3-monooxygenase
BCAM2116	2.17	conserved hypothetical protein
BCAM2138	2.58	conserved hypothetical protein
BCAM2139	2.36	conserved hypothetical protein
BCAM2149	2.08	metallo peptidase, subfamily M20A
BCAM2212	2.38	2Fe-2S iron-sulfur

Gene name	Fold change	Annotation
BCAM2393	2.51	AraC family regulatory protein
BCAM2401	2.21	putative aspartyl/asparaginyl beta-hydroxylase
BCAM2413	7.46	putative GNAT family N-acetyltransferase
BCAM2414	2.90	conserved hypothetical protein
BCAM2417	2.01	conserved hypothetical protein
BCAM2420	2.50	conserved hypothetical protein
BCAM2421	2.90	conserved hypothetical protein
BCAM2429	2.91	putative phospholipase C
BCAM2429a	3.52	putative lipoprotein
BCAM2500	3.88	putative glucarate transporter
BCAM2501	2.86	shikimate 5-dehydrogenase
BCAM2502	4.59	3-dehydroquinate dehydratase
BCAM2580	2.84	putative tartrate transporter
BCAM2686	2.27	putative membrane protein
BCAM2720	6.85	putative phospholipase C
BCAM2721	2.53	conserved hypothetical protein
BCAM2773	2.15	putative DNA-binding protein
BCAM2803	2.22	putative stress-induced protein
BCAM2808	2.04	putative extracellular solute-binding protein
BCAS0062	2.69	LysR family regulatory protein
BCAS0065	2.13	putative glutathione S-transferase
BCAS0115	2.28	GntR family regulatory protein
BCAS0188a	2.34	hypothetical protein
BCAS0189	2.14	conserved hypothetical protein
BCAS0193	2.09	putative dehydrogenase
BCAS0242	4.50	conserved hypothetical protein
BCAS0244	14.47	hypothetical protein
BCAS0245	15.24	30S ribosomal protein S21 3
BCAS0246	11.59	conserved hypothetical protein
BCAS0247	6.45	hypothetical protein
BCAS0249	2.27	chromate resistance transport protein
BCAS0262	2.53	putative acetyltransferase
BCAS0321b	2.26	hypothetical protein
BCAS0368	2.09	citrate utilization protein B
BCAS0382	2.18	AnsC family regulatory protein
BCAS0517	2.27	putative phage tail tube protein
BCAS0518	2.60	putative phage tail sheath protein
BCAS0532	2.08	putative phage exported protein Rz
BCAS0684_J_0	2.33	conserved hypothetical protein (fragment)
BCAS0688	2.36	TetR family regulatory protein
BCAS0697	2.10	LysR family regulatory protein
BCAS0721	2.09	conserved hypothetical protein
BCAS0722	2.13	putative patatin-like phospholipase
BCAS0748	2.02	putative calcineurin-like phosphoesterase family
BCAS0755	2.87	putative membrane protein
pBCA029	2.54	putative membrane protein

Gene name	Fold change	Annotation
pBCA053	2.43	putative extracellular solute-binding protein
pBCA054	2.19	LuxR family regulatory protein
IG1_1241336	2.56	interG_chr1_pos_682_1241336:1241457
IG1_1598102	2.14	interG_chr1_pos_869_1598102:1602124
IG1_1774432	2.10	interG_chr1_pos_956_1774432:1775333
IG1_2151274	2.26	interG_chr1_pos_1124_2151274:2151732
IG1_2379897	4.00	interG_chr1_pos_1211_2379897:2382130
IG1_2510004	3.25	interG_chr1_pos_1248_2510004:2511399
IG1_2538832	2.07	interG_chr1_pos_1253_2538832:2538943
IG1_2575094	2.26	interG_chr1_pos_1276_2575094:2575991
IG1_2620209	2.29	interG_chr1_pos_1288_2620209:2620409
IG1_2653432	6.10	interG_chr1_pos_1304_2653432:2653684
IG1_2657870	2.79	interG_chr1_pos_1308_2657870:2669736
IG1_2696183	2.46	interG_chr1_pos_1322_2696183:2696350
IG1_2941513	2.40	interG_chr1_pos_1447_2941513:2941640
IG1_3070378	2.16	interG_chr1_pos_1507_3070378:3070549
IG1_3126984	2.10	interG_chr1_pos_1523_3126984:3128293
IG1_3241588	2.43	interG_chr1_pos_1566_3241588:3241750
IG1_3543041	2.12	interG_chr1_pos_1726_3543041:3543103
IG1_3567797	2.16	interG_chr1_pos_1734_3567797:3569631
IG1_360976	2.06	interG_chr1_pos_238_360976:362112
IG1_3716487	2.02	interG_chr1_pos_1798_3716487:3716574
IG1_3865646	2.06	interG_chr1_pos_1841_3865646:3865810
IG1_42625	2.26	interG_chr1_pos_32_42625:44320
IG1_486377	2.63	interG_chr1_pos_301_486377:487637
IG1_489810	3.40	interG_chr1_pos_302_489810:490004
IG1_721602	2.03	interG_chr1_pos_412_721602:732050
IG1_765386	2.10	interG_chr1_pos_425_765386:765539
IG1_958289	2.35	interG_chr1_pos_536_958289:958488
IG2_1103454	2.60	interG_chr2_pos_571_1103454:1103560
IG2_1159867	2.79	interG_chr2_pos_597_1159867:1159974
IG2_1175538	2.29	interG_chr2_pos_618_1175538:1175673
IG2_1754913	4.08	interG_chr2_pos_896_1754913:1757611
IG2_1823152	2.72	interG_chr2_pos_918_1823152:1823601
IG2_1882664	2.02	interG_chr2_pos_938_1882664:1882783
IG2_1899056	2.00	interG_chr2_pos_945_1899056:1899370
IG2_1926503	6.90	interG_chr2_pos_955_1926503:1926918
IG2_2012657	2.38	interG_chr2_pos_999_2012657:2013879
IG2_2029035	5.43	interG_chr2_pos_1007_2029035:2029415
IG2_2050164	4.83	interG_chr2_pos_1019_2050164:2050639
IG2_2086694	2.04	interG_chr2_pos_1033_2086694:2087541
IG2_2447127	2.09	interG_chr2_pos_1188_2447127:2448873
IG2_2586922	4.63	interG_chr2_pos_1237_2586922:2587412
IG2_2731412	3.04	interG_chr2_pos_1296_2731412:2731800
IG2_3132121	2.22	interG_chr2_pos_1451_3132121:3133304

Gene name	Fold change	Annotation
IG2_321816	2.10	interG_chr2_pos_144_321816:323991
IG2_551370	3.66	interG_chr2_pos_283_551370:553896
IG2_683264	4.57	interG_chr2_pos_355_683264:683909
IG2_794541	2.54	interG_chr2_pos_404_794541:797119
IG3_264432	2.41	interG_chr3_pos_143_264432:264707
IG3_278948	2.35	interG_chr3_pos_148_278948:280854
IG3_536263	2.59	interG_chr3_pos_259_536263:537450
IG3_663568	2.30	interG_chr3_pos_305_663568:669511
IG3_811123	3.17	interG_chr3_pos_363_811123:815031
BCAL2508	2.09	intergenic region between BCAL2507 and BCAL2509
