# Supplementary material for: Transcriptional response of Burkholderia cenocepacia J2315 sessile cells to treatments with high doses of hydrogen peroxide and sodium hypochlorite
Source: BMC Genomics. 2010 Feb 5;11:90. doi: 10.1186/1471-2164-11-90 (PMC2830190; doi:10.1186/1471-2164-11-90)
Supplement: Additional file 3 — Upregulated genes, intergenic regions and tRNA- and rRNA- encoding sequences in NaOCl-treated biofilms. Complete list of all B. cenocepacia J2315 genes, intergenic regions and tRNA- and rRNA- encoding sequences showing a significantly increased expression (>2-fold change; p < 0.05) in NaOCl-treated biofilms compared to the expression in the untreated biofilms. [file 1471-2164-11-90-S3.PDF]

Gene name	Fold change	Annotation
BCAL0006	2.33	putative protein lysine methyltransferase
BCAL0020	2.16	putative branched-chain amino acid ABC
BCAL0029	2.97	putative ATP synthase protein I AtpI
BCAL0031	3.30	ATP synthase C chain
BCAL0032	3.26	ATP synthase B chain
BCAL0033	2.54	ATP synthase delta chain
BCAL0034	2.92	ATP synthase alpha chain
BCAL0035	2.31	ATP synthase gamma chain
BCAL0042	11.28	bifunctional PutA protein [includes: proline
BCAL0074	2.05	glycine cleavage system H protein
BCAL0076	2.08	putative lipoprotein
BCAL0118	2.35	DNA-3-methyladenine glycosylase I
BCAL0123	2.50	putative glycosyltransferase
BCAL0125	2.04	flagellar regulon master regulator subunit FlhC
BCAL0126	3.26	chemotaxis protein MotA
BCAL0145	2.38	adenosylhomocysteinase
BCAL0154	2.46	histone-like nucleoid-structuring (H-NS)
BCAL0155	2.75	putative cation efflux protein
BCAL0206A	3.27	putative outer membrane protein
BCAL0219	2.44	elongation factor Tu
BCAL0230	2.09	30S ribosomal protein S7
BCAL0232	2.32	elongation factor Tu (EF-Tu)
BCAL0249	2.56	50S ribosomal protein L6
BCAL0254	2.15	preprotein translocase SecY subunit
BCAL0260	2.10	DNA-directed RNA polymerase alpha chain
BCAL0261	2.97	50S ribosomal protein L17
BCAL0263	2.65	thiol:disulfide interchange protein DsbD
BCAL0264	2.29	delta-aminolevulinic acid dehydratase
BCAL0269	2.12	putative oxidoreductase
BCAL0291	3.46	sodium:amino acid symporter family protein
BCAL0305	2.11	putative exported protein
BCAL0340	2.56	putative lipoprotein
BCAL0364	3.70	TetR family regulatory protein
BCAL0365	4.95	Major Facilitator Superfamily protein
BCAL0367	2.39	putative chaperone protein
BCAL0369	2.36	putative amino acid permease
BCAL0388	2.23	putative monooxygenase
BCAL0412	2.24	conserved hypothetical protein (pseudogene)
BCAL0413	2.14	conserved hypothetical protein
BCAL0426	3.13	putative membrane protein
BCAL0439	2.01	glutamate--cysteine ligase
BCAL0455	2.84	putative hydrolase
BCAL0468	4.22	metallo peptidase, subfamily M48B

Gene name	Fold change	Annotation
BCAL0474	2.11	conserved hypothetical protein
BCAL0482	2.80	putative rod shape-determining protein
BCAL0496	2.04	putative acetylglutamate kinase
BCAL0509	2.55	S-adenosylmethionine synthetase
BCAL0510	2.59	conserved hypothetical protein
BCAL0527	3.36	flagellar protein FliS
BCAL0528	2.81	conserved hypothetical protein
BCAL0567	2.71	flagellar hook protein 1 FlgE1
BCAL0568	2.38	flagellar basal-body rod protein FlgF (putative
BCAL0575	2.96	YcgR family protein
BCAL0576	4.70	flagellar hook-associated protein 1 (HAP1)
BCAL0577	4.30	flagellar hook-associated protein 3 (HAP3)
BCAL0591	3.33	putative transposase
BCAL0597	2.15	putrescine ABC transporter ATP-binding protein
BCAL0600	2.12	putative glutamine synthetase
BCAL0605	2.19	putative exported protein
BCAL0655	2.34	CutC family protein
BCAL0677	3.44	thiol:disulfide interchange protein
BCAL0722	6.17	C4-dicarboxylate transport protein
BCAL0762	3.51	putative methyl-accepting chemotaxis protein
BCAL0763	5.40	putative exported protein
BCAL0783	3.91	putative membrane protein
BCAL0784	3.74	cytochrome d ubiquinol oxidase subunit II
BCAL0785	4.82	cytochrome d ubiquinol oxidase subunit I
BCAL0786	4.22	putative membrane protein
BCAL0787	2.62	RNA polymerase σ^{32}-factor
BCAL0796	2.09	ferredoxin
BCAL0830	2.39	putative ParA family protein
BCAL0831	3.79	putative storage protein
BCAL0833	5.53	putative Acetoacetyl-CoA reductase
BCAL0849	2.54	metallo peptidase, subfamily M48B
BCAL0899	4.89	lactoylglutathione lyase
BCAL0934	2.59	putative periplasmic cytochrome c containing
BCAL0947	2.42	putative membrane protein
BCAL0960	2.62	O-antigen polymerase family protein
BCAL0996	2.05	3-oxoacyl-[acyl-carrier-protein] synthase II 2
BCAL1031	2.24	aetyltransferase (GNAT) family protein
BCAL1033	2.85	putative exported protein
BCAL1034	2.15	SCO1/SenC family protein
BCAL1040	2.71	glycosyl transferases group 1 protein
BCAL1067	3.17	putative transposase
BCAL1071	2.81	NAD dependent epimerase/dehydratase family
BCAL1091	2.27	ABC transporter membrane protein
BCAL1092	3.10	ABC transporter extracellular solute-binding
BCAL1093	2.32	putative membrane protein
BCAL1103	3.14	putative OsmB-like lipoprotein

Gene name	Fold change	Annotation
BCAL1105	12.65	putative exported protein
BCAL1106	8.31	cytochrome b561 family protein
BCAL1107	3.42	putative oxidoreductase
BCAL1236	2.91	putative exported protein
BCAL1251	4.83	conserved hypothetical protein
BCAL1263	3.87	transcription elongation factor
BCAL1270	2.98	phosphate transport system, substrate-binding
BCAL1354	2.77	conserved hypothetical protein
BCAL1368	2.52	putative porin
BCAL1411	2.57	putative exported protein
BCAL1412	2.53	NUDIX hydrolase
BCAL1425	2.45	putative glucose 1-dehydrogenase
BCAL1439	2.39	methylmalonic acid semialdehyde dehydrogenase
BCAL1475	2.47	putative polysaccharide deacetylase
BCAL1488	2.12	MerR family regulatory protein
BCAL1516	2.08	dihydrolipoamide succinyltransferase component
BCAL1518	2.05	AFG1-like ATPase
BCAL1556	2.07	ribose 5-phosphate isomerase A
BCAL1635a	5.68	putative exported protein
BCAL1638	2.81	thioesterase superfamily protein
BCAL1662	3.08	putative methyl-accepting chemotaxis protein
BCAL1666	2.48	alpha-ketoglutarate permease
BCAL1667	2.47	hypothetical protein
BCAL1674	4.93	multidrug efflux system AmrA protein
BCAL1717A	2.10	putative exported protein
BCAL1733	2.13	putative glutathione S-transferase
BCAL1752	2.89	conserved hypothetical protein
BCAL1763	30.15	putative exported protein
BCAL1764	15.32	putative exported protein
BCAL1765	31.00	putative exported protein
BCAL1766	75.22	OsmC-like protein
BCAL1808	5.92	putative membrane protein
BCAL1822	2.62	putrescine transport system permease protein
BCAL1823	2.42	putrescine ABC transporter ATP-binding protein
BCAL1824	5.21	putrescine-binding periplasmic protein
BCAL1829	7.90	putative outer membrane protein
BCAL1830	11.63	putative 2-nitropropane dioxygenase
BCAL1939	3.28	putative integral membrane transport protein
BCAL1961	2.50	putative exported protein
BCAL1992	2.27	putative acyl-CoA thioesterase precursor
BCAL2007	2.42	squalene/phytoene synthase family protein
BCAL2014	3.26	carboxymuconolactone decarboxylase family
BCAL2015	2.03	2-C-methyl-D-erythritol 2,4-cyclodiphosphate
BCAL2044	3.81	muramoyltetrapeptide carboxypeptidase
BCAL2075	2.21	putative ATP-binding protein
BCAL2123	3.30	conserved hypothetical protein

Gene name	Fold change	Annotation
BCAL2127	2.26	putative exported phospholipase
BCAL2128	3.25	cysteine peptidase, family C40
BCAL2141	2.20	cytochrome O ubiquinol oxidase protein
BCAL2143	4.12	ubiquinol oxidase polypeptide I
BCAL2144	2.79	ubiquinol oxidase polypeptide II precursor
BCAL2191	2.56	putative membrane protein
BCAL2206	2.84	phasin-like protein
BCAL2288	2.10	bacterioferritin
BCAL2349	2.44	30S ribosomal protein S15
BCAL2350	2.12	putative amino acid transporter exported
BCAL2352	2.22	putative carbonic anhydrase
BCAL2353	2.19	putative sulfate transporter
BCAL2356	2.73	putative decarboxylase
BCAL2428	3.10	putative cytochrome C precursor-related protein
BCAL2429	3.22	putative cytochrome C precursor-related protein
BCAL2437	3.80	conserved hypothetical protein
BCAL2442	4.82	chaperone protein HtpG
BCAL2455	2.54	topoisomerase IV subunit B
BCAL2467	2.47	putative lipoprotein
BCAL2468	2.04	putative membrane protein
BCAL2472	2.76	alpha,alpha-trehalose-phosphate synthase
BCAL2500	2.25	hypothetical protein
BCAL2523A	2.33	putative membrane protein
BCAL2545	2.02	Major Facilitator Superfamily protein
BCAL2558	2.11	putative thioredoxin/FAD-dependent pyridine
BCAL2559	2.18	putative transcriptional regulator
BCAL2609	3.21	periplasmic histidine-binding protein
BCAL2610	2.53	histidine transport system permease protein
BCAL2612	2.49	histidine ABC transporter ATP-binding protein
BCAL2641	2.43	putative ornithine decarboxylase
BCAL2652	2.25	putative aspartate 1-decarboxylase
BCAL2667	2.21	cell division protein ZapA
BCAL2685	7.34	putative sulfite reductase
BCAL2703	4.23	conserved hypothetical protein
BCAL2719	2.28	putative transmembrane fatty acid desaturase
BCAL2791	2.10	putative kynureninase
BCAL2813	2.34	putative periplasmic solute-binding protein
BCAL2838	2.70	5'-phosphoribosyl-4-N-succinocarboxamide-5-amin
BCAL2874	2.09	3-oxoacyl-[acyl-carrier-protein] synthase II 1
BCAL2899	3.88	4Fe-4S ferredoxin
BCAL2933	2.30	D-amino acid dehydrogenase small subunit
BCAL2941	2.18	putative exported transglycosylase
BCAL2949	2.16	integration host factor beta-subunit
BCAL2987	2.56	disulfide bond formation protein B
BCAL3006	2.42	cold shock-like protein
BCAL3026	2.56	septum site-determining protein

Gene name	Fold change	Annotation
BCAL3033	2.23	probable outer-membrane lipoproteins carrier
BCAL3058	2.39	3-octaprenyl-4-hydroxybenzoate carboxy-lyase
BCAL3076A	2.12	putative DNA-binding protein
BCAL3094	2.47	oxygen-independent coproporphyrinogen III
BCAL3111	2.72	conserved hypothetical protein
BCAL3146	2.92	60 kDa chaperonin 1
BCAL3147	4.45	10 kDa chaperonin 1
BCAL3200	2.69	putative TolQ transport transmembrane protein
BCAL3214	35.05	carboxymuconolactone decarboxylase family
BCAL3215	3.48	cysteine peptidase, family C44
BCAL3227	2.77	conserved hypothetical protein
BCAL3228	2.30	hypothetical protein
BCAL3231	2.07	hypothetical protein
BCAL3235	2.49	putative UDP-galactopyranose mutase
BCAL3239	2.04	glucosyltransferase
BCAL3243	2.99	putative capsular polysaccharide
BCAL3285	10.14	flavohemoprotein
BCAL3305	2.64	preprotein translocase subunit
BCAL3307	2.75	putative protein-export membrane protein
BCAL3310	2.63	putative exported protein
BCAL3311	3.27	putative exported protein
BCAL3359	2.96	putative glutamate dehydrogenase
BCAL3364	2.67	putative gluconokinase
BCAL3393	2.11	conserved hypothetical protein
BCAL3420	2.46	biotin carboxyl carrier protein of acetyl-CoA
BCAL3421	2.15	biotin carboxylase
BCAL3425	2.09	putative sugar kinase
BCAL3438	2.54	putative lipoprotein
BCAL3473	4.97	putative outer membrane porin
BCAL3492	2.80	putative exported protein
BCAL3494	3.39	type III restriction-modification system
BCAL3514	3.10	outer membrane efflux protein
BCAM0010	2.40	2-amino-3-ketobutyrate coenzyme A ligase
BCAM0011	3.27	threonine 3-dehydrogenase
BCAM0042	2.54	putative aldo/keto reductase
BCAM0043	2.23	conserved hypothetical protein
BCAM0050	2.15	universal stress-related protein
BCAM0163	3.07	putative microcin immunity protein
BCAM0165	5.47	conserved hypothetical protein
BCAM0275a	2.82	conserved hypothetical protein
BCAM0276	4.01	putative universal stress protein
BCAM0277	3.26	conserved hypothetical protein
BCAM0278	9.23	putative heat shock protein
BCAM0280	8.55	putative phospholipid-binding protein
BCAM0280A	4.13	conserved hypothetical protein
BCAM0284	4.45	putative cytochrome c

Gene name	Fold change	Annotation
BCAM0285	4.26	conserved hypothetical protein
BCAM0286	2.12	putative alcohol dehydrogenase
BCAM0290	4.36	putative universal stress protein
BCAM0291	5.37	putative universal stress protein
BCAM0292	5.43	putative universal stress protein
BCAM0293	4.24	putative acetate kinase
BCAM0294	7.15	putative universal stress protein
BCAM0295	8.04	conserved hypothetical protein
BCAM0296	5.04	acetoacetyl-CoA reductase
BCAM0297	5.08	putative polymerase
BCAM0298	2.96	putative phosphate acetyl/butyryl transferase
BCAM0299	3.39	putative zinc-binding alcoholdehydrogenase
BCAM0300	3.64	metallo-beta-lactamase superfamily protein
BCAM0303	2.82	ABC transporter ATP-binding membrane protein
BCAM0305	2.84	outer membrane transport system protein
BCAM0306	5.43	putative membrane protein
BCAM0307	5.59	conserved hypothetical protein
BCAM0308	7.13	conserved hypothetical protein
BCAM0309	5.18	putative cell division-related metallo
BCAM0311	10.40	putative 6-phosphofructokinase
BCAM0312	4.98	putative polysaccharide deacetylase
BCAM0313	2.90	putative exported protein
BCAM0316	3.20	conserved hypothetical protein
BCAM0317	2.76	putative membrane protein
BCAM0320	3.60	putative cytochrome b561
BCAM0321	2.75	conserved hypothetical protein
BCAM0343	2.38	putative redoxin
BCAM0374	3.00	conserved hypothetical protein
BCAM0383	2.39	putative lipoprotein
BCAM0384	2.66	putative lipoprotein
BCAM0548	3.56	60 kDa chaperonin 2
BCAM0588	2.62	MarR family regulatory protein
BCAM0598	3.08	putative reductase
BCAM0674	6.01	putative dehydrogenase
BCAM0750	2.25	conserved hypothetical protein
BCAM0776	2.68	putative cNMP-binding domain protein
BCAM0778	2.30	OmpA family protein
BCAM0810	2.74	putative aromatic oxygenase
BCAM0896	37.16	putative organic hydroperoxide resistance
BCAM0906	2.46	putative dienelactone hydrolase family protein
BCAM0916	3.00	conserved hypothetical protein
BCAM0944	2.29	putative lipoprotein
BCAM1003	2.32	putative epimerase
BCAM1015	2.97	putative porin
BCAM1061	3.79	hypothetical phage protein
BCAM1062	2.68	hypothetical phage protein

Gene name	Fold change	Annotation
BCAM1063	2.52	hypothetical phage protein
BCAM1111	6.24	ornithine decarboxylase
BCAM1113	3.31	putrescine transport protein
BCAM1200	2.61	putative membrane protein
BCAM1204	2.05	alanine racemase, catabolic
BCAM1216	2.12	alkyl hydroperoxide reductase subunit F
BCAM1217	15.03	alkyl hydroperoxide reductase subunit C
BCAM1219	4.36	putative ligand-binding receptor
BCAM1220	3.87	putative hydrolase
BCAM1221	2.23	putative amidohydrolase/peptidase
BCAM1222	2.28	putative cytochrome
BCAM1242A	2.48	putative exported protein
BCAM1290	2.41	RpiR-family transcriptional regulator
BCAM1316a	2.34	conserved hypothetical protein
BCAM1316b	2.78	conserved hypothetical protein
BCAM1354	2.96	putative membrane protein
BCAM1398	3.23	putative porin
BCAM1407	2.16	DJ-1/PfpI family protein
BCAM1421	2.95	RND family efflux system transporter protein
BCAM1424	3.44	methyl-accepting chemotaxis protein
BCAM1433	4.13	putative short chain dehydrogenase
BCAM1481	2.35	conserved hypothetical protein
BCAM1482	2.54	conserved hypothetical protein
BCAM1492	2.45	putative exported protein
BCAM1496	2.49	conserved hypothetical protein
BCAM1500	2.15	putative universal stress protein
BCAM1502	5.90	conserved hypothetical protein
BCAM1503	5.66	putative methyl-accepting chemotaxis protein
BCAM1537	2.03	putative dehydrogenase, zinc-binding subunit
BCAM1570	4.74	alcohol dehydrogenase
BCAM1571	2.24	TonB-dependent receptor
BCAM1572	4.10	methyl-accepting chemotaxis protein
BCAM1606	2.04	electron transfer flavoprotein, alpha subunit
BCAM1652	2.66	putative lipoprotein
BCAM1727	2.57	conserved hypothetical protein
BCAM1744	5.70	serine peptidase, family S9
BCAM1754	4.68	putative mechanosensitive ion channel
BCAM1775	2.34	putative transglycosylase associated protein
BCAM1776	2.29	putative transposase
BCAM1777A	3.39	putative exported protein
BCAM1780	3.94	peptidoglycan-binding lysm:peptidase m23b
BCAM1787	2.44	putative porin
BCAM1804	10.34	methyl-accepting chemotaxis protein
BCAM1833	2.14	aconitate hydratase
BCAM1911	2.19	hypothetical phage protein
BCAM1912	4.32	hypothetical phage protein

Gene name	Fold change	Annotation
BCAM1914	2.02	hypothetical phage protein (pseudogene)
BCAM1923	2.39	phage integrase (pseudogene)
BCAM1931	2.17	putative porin
BCAM1933	2.20	putative cyclase
BCAM1954	2.31	sodium:dicarboxylate symporter
BCAM2006	5.55	putative aspartate carbomyltransferase
BCAM2021	3.17	methyl-accepting chemotaxis protein
BCAM2073	2.66	putative exported protein
BCAM2151	2.72	conserved hypothetical rhodanese family protein
BCAM2159	2.25	putative exported protein
BCAM2176	2.66	putative thermolabile glutaminase
BCAM2246	2.05	LysR family regulatory protein
BCAM2341	2.27	TetR family regulatory protein
BCAM2342	2.90	putative betaine aldehyde dehydrogenase
BCAM2377	2.41	putative exported protein
BCAM2395	4.79	putative dehydrogenase/oxidoreductase protein
BCAM2396	4.50	conserved hypothetical protein
BCAM2397	4.97	putative L-fuculose phosphate aldolase
BCAM2457	2.60	putative exported protein
BCAM2479	4.83	putative transporter - LysE family
BCAM2483	2.00	Major Facilitator Superfamily protein
BCAM2536	3.00	putative alpha-beta hydrolase
BCAM2537	2.33	putative membrane protein
BCAM2545	6.32	Major Facilitator Superfamily protein
BCAM2546	3.65	putative MlrC family protein
BCAM2552	4.93	putative hydrolase
BCAM2562	2.25	putative succinate-semialdehyde dehydrogenase
BCAM2563	2.20	methyl-accepting chemotaxis protein
BCAM2564	3.74	putative aerotaxis receptor
BCAM2584	4.26	putative gram-negative porin
BCAM2594	2.49	putative alcohol dehydrogenase
BCAM2595	2.21	putative carboxymuconolactone dehydrogenase
BCAM2609	2.62	putative exported protein
BCAM2615	2.26	putative membrane protein
BCAM2620	5.85	putative lipoprotein
BCAM2621_J_0	3.37	putative porin-related protein (pseudogene)
BCAM2621_J_1	3.25	putative porin-related protein (pseudogene)
BCAM2637	3.31	putative transposase
BCAM2639	2.34	putative membrane protein
BCAM2640A	2.30	putative membrane protein
BCAM2644	2.44	putative glutathione S-transferase
BCAM2650	4.24	putative short-chain dehydrogenase/reductase
BCAM2677	4.55	putative membrane protein
BCAM2689	2.21	putative methyl-accepting chemotaxis protein
BCAM2705	2.47	conserved hypothetical protein
BCAM2709	2.54	conserved hypothetical protein

Gene name	Fold change	Annotation
BCAM2813	2.31	conserved hypothetical protein
BCAM2836	2.11	putative diguanylate cyclase
BCAM2837_J_0	2.36	two-component regulatory system, response
BCAM2837_J_1	3.71	two-component regulatory system, response
BCAS0081	2.52	ABC transporter ATP-binding membrane protein
BCAS0084	3.84	TetR family regulatory protein
BCAS0085	20.28	organic hydroperoxide resistance protein
BCAS0086	11.27	putative lipase
BCAS0118	2.64	putative H-NS family DNA-binding protein
BCAS0151	2.18	hypothetical protein
BCAS0251	2.67	putative lipoprotein
BCAS0258	2.05	GntR family regulatory protein
BCAS0398	2.32	putative diguanylate cyclase
BCAS0637	2.75	60 kDa chaperonin 3
BCAS0638	4.43	10 kDa chaperonin 3
BCAS0648	2.56	hypothetical protein
BCAS0677	2.49	conserved hypothetical protein
BCAS0744	2.07	conserved hypothetical protein
pBCA001	2.24	putative partition protein
pBCA064	2.13	putative membrane protein
pBCA087	3.87	NUDIX hydrolase family protein
IG1_1014163	3.76	interG_chr1_pos_558_1014163:1014489
IG1_1125732	2.34	interG_chr1_pos_626_1125732:1125966
IG1_1154931	3.89	interG_chr1_pos_645_1154931:1155285
IG1_1211174	4.37	interG_chr1_pos_663_1211174:1211249
IG1_1237542	3.91	interG_chr1_pos_680_1237542:1240205
IG1_1374638	2.25	interG_chr1_pos_744_1374638:1374755
IG1_1556876	2.69	interG_chr1_pos_841_1556876:1556984
IG1_1884990	2.42	interG_chr1_pos_1024_1884990:1885313
IG1_2102402	2.23	interG_chr1_pos_1103_2102402:2102557
IG1_2221074	2.11	interG_chr1_pos_1150_2221074:2221262
IG1_2420168	2.47	interG_chr1_pos_1223_2420168:2420460
IG1_2646467	2.12	interG_chr1_pos_1296_2646467:2646584
IG1_288839	2.49	interG_chr1_pos_174_288839:288976
IG1_2935724	4.65	interG_chr1_pos_1444_2935724:2936297
IG1_2936760	2.97	interG_chr1_pos_1445_2936760:2936941
IG1_300118	2.66	interG_chr1_pos_182_300118:300511
IG1_3008003	2.42	interG_chr1_pos_1476_3008003:3008659
IG1_3114330	2.19	interG_chr1_pos_1521_3114330:3124273
IG1_3139352	5.18	interG_chr1_pos_1527_3139352:3139618
IG1_3180283	5.36	interG_chr1_pos_1537_3180283:3180384
IG1_3286080	2.75	interG_chr1_pos_1601_3286080:3286285
IG1_3322060	2.25	interG_chr1_pos_1625_3322060:3327025
IG1_3492560	2.23	interG_chr1_pos_1701_3492560:3495240
IG1_3537906	2.22	interG_chr1_pos_1722_3537906:3539109

Gene name	Fold change	Annotation
IG1_3556424	3.48	interG_chr1_pos_1730_3556424:3556621
IG1_3627057	2.44	interG_chr1_pos_1763_3627057:3630580
IG1_37663	3.30	interG_chr1_pos_28_37663:37730
IG1_3822505	3.40	interG_chr1_pos_1830_3822505:3822812
IG1_3828501	2.25	interG_chr1_pos_1835_3828501:3828687
IG1_422927	4.04	interG_chr1_pos_269_422927:423016
IG1_435852	2.24	interG_chr1_pos_275_435852:436014
IG1_52439	2.44	interG_chr1_pos_35_52439:52522
IG1_687802	2.45	interG_chr1_pos_396_687802:688965
IG1_825611	5.91	interG_chr1_pos_472_825611:825836
IG1_868117	2.14	interG_chr1_pos_490_868117:868227
IG1_901672	16.17	interG_chr1_pos_504_901672:902168
IG1_950309	2.31	interG_chr1_pos_528_950309:950472
IG2_1126199	2.15	interG_chr2_pos_582_1126199:1126466
IG2_1423308	2.31	interG_chr2_pos_737_1423308:1423409
IG2_1660910	2.74	interG_chr2_pos_846_1660910:1661218
IG2_1669623	2.04	interG_chr2_pos_852_1669623:1670980
IG2_1953120	3.21	interG_chr2_pos_965_1953120:1953814
IG2_1975919	2.36	interG_chr2_pos_977_1975919:1976205
IG2_2085682	4.33	interG_chr2_pos_1032_2085682:2085985
IG2_208795	2.44	interG_chr2_pos_100_208795:209162
IG2_2970010	2.64	interG_chr2_pos_1386_2970010:2971563
IG2_3145193	2.00	interG_chr2_pos_1456_3145193:3145405
IG2_366910	2.25	interG_chr2_pos_166_366910:367027
IG2_369070	2.66	interG_chr2_pos_168_369070:370084
IG2_429106	4.49	interG_chr2_pos_211_429106:432827
IG2_433416	4.09	interG_chr2_pos_212_433416:433537
IG2_746545	36.45	interG_chr2_pos_380_746545:746842
IG2_747590	3.37	interG_chr2_pos_381_747590:747741
BCAL0181	2.62	intergenic region between BCAL0180 and BCAL0181
BCAL1136	4.73	intergenic region between BCAL1132 and BCAL1138
BCAL1137	2.17	intergenic region between BCAL1132 and BCAL1138
BCALr1899	2.75	Bacterial signal recognition particle RNA (RF00169)
BCALr0080	8.18	tRNA Arg anticodon CCG, Cove score 82.41
BCALr0218c	2.64	tRNA Thr anticodon GGT, Cove score 90.12
BCALr0472	3.30	tRNA Phe anticodon GAA, Cove score 86.97
BCALr0949	3.22	tRNA Met anticodon CAT, Cove score 88.36
BCALr0970a	3.23	tRNA Asn anticodon GTT, Cove score 85.87
BCALr1279	2.95	tRNA Pro anticodon TGG, Cove score 89.00
BCALr1551a	3.62	tRNA Leu anticodon CAG, Cove score 72.78
BCAMr0918	2.75	tRNA Met anticodon CAT, Cove score 71.40
BCASr0743b	2.55	23 S RNA
