# Supplementary material for: Transcriptional response of Burkholderia cenocepacia J2315 sessile cells to treatments with high doses of hydrogen peroxide and sodium hypochlorite
Source: BMC Genomics. 2010 Feb 5;11:90. doi: 10.1186/1471-2164-11-90 (PMC2830190; doi:10.1186/1471-2164-11-90)
Supplement: Additional file 4 — Downregulated genes, intergenic regions and tRNA- and rRNA- encoding sequences in NaOCl-treated biofilms. Complete list of all B. cenocepacia J2315 genes, intergenic regions and tRNA- and rRNA- encoding sequences showing a significantly decreased expression (>2-fold change; p < 0.05) in NaOCl-treated biofilms compared to the expression in the untreated biofilms. [file 1471-2164-11-90-S4.PDF]

Gene name	Fold change	Annotation
BCAL0008A	2.15	conserved hypothetical protein
BCAL0046	2.90	putative fatty-acid CoA ligase
BCAL0047	2.87	putative acyl-CoA dehydrogenase
BCAL0121	8.26	aquaporin Z
BCAL0122	2.43	histone-like nucleoid-structuring (H-NS)
BCAL0169	2.18	conserved hypothetical protein
BCAL0209	2.87	acetyltransferase (GNAT) family protein
BCAL0272	2.08	putative lipoprotein
BCAL0418	2.28	type I restriction enzyme specificity protein
BCAL0431	6.90	conserved hypothetical protein
BCAL0432	7.94	putative membrane protein
BCAL0444	4.85	GntR family regulatory protein
BCAL0516	2.74	conserved hypothetical protein
BCAL0518	2.39	putative coniferyl aldehyde dehydrogenase
BCAL0530	2.08	putative export system protein
BCAL0539	3.03	putative nitroreductase/p-nitrobenzoate
BCAL0554	2.67	5-formyltetrahydrofolate cyclo-ligase family
BCAL0617	2.34	conserved hypothetical protein
BCAL0625	12.45	LysR family regulatory protein
BCAL0627	2.04	putative hydrolase
BCAL0665	4.52	dethiobiotin synthetase
BCAL0709	2.11	putative lipoate-protein ligase B
BCAL0744	3.30	Appr-1-p processing enzyme family protein
BCAL0745	2.17	SpoU rRNA methylase family protein
BCAL0755	2.44	putative membrane protein
BCAL0759	2.13	UbiA prenyltransferase family protein
BCAL0768	2.18	conserved hypothetical protein
BCAL0777	4.85	GntR family regulatory protein
BCAL0778	5.38	putative N-acetylglucosamine-6-phosphate
BCAL0779	5.35	putative phosphosugar-binding protein
BCAL0780	3.80	putative multiphosphoryl transfer protein
BCAL0781	4.12	phosphotransferase system, IIbc component
BCAL0804	2.22	putative membrane protein
BCAL0813	3.13	putative RNA polymerase σ^{54}-factor
BCAL0857	2.10	putative exported protein
BCAL0859	2.55	putative hydrolase
BCAL0861	2.27	Major Facilitator Superfamily protein
BCAL0864	2.09	conserved hypothetical protein
BCAL0883	2.92	TetR family regulatory protein
BCAL0907	2.09	putative cation transporter efflux protein
BCAL0950	7.19	Major Facilitator Superfamily protein
BCAL0961	3.60	putative lipoprotein
BCAL1023	4.61	putative DNA-binding protein

Gene name	Fold change	Annotation
BCAL1028_J_0_1	2.21	putative transposase (pseudogene)
BCAL1028_J_1	2.02	putative transposase (pseudogene)
BCAL1044	2.78	GntR family regulatory protein
BCAL1099	2.02	putative release factor
BCAL1161	2.65	conserved hypothetical protein
BCAL1163	2.06	putative transposase
BCAL1176	2.08	putative fusaric acid resistance transport
BCAL1182	2.11	putative transcriptional regulator
BCAL1257	2.66	putative gultathione hydrolase
BCAL1277	2.08	polyphosphate kinase
BCAL1279	2.15	putative exported protein
BCAL1380	4.93	(R)-stereoselective amidase
BCAL1442	2.54	conserved hypothetical protein
BCAL1444	2.07	putative exported protein
BCAL1464	2.96	putative membrane protein
BCAL1478	2.26	putative hydrolase
BCAL1490	2.43	putative exported protein
BCAL1492	2.62	conserved hypothetical protein
BCAL1498	3.22	conserved hypothetical protein
BCAL1519	2.05	putative transposase
BCAL1520	2.53	putative lipoprotein
BCAL1535	2.00	putative membrane protein
BCAL1622	3.42	molybdenum-pterin binding protein II
BCAL1623	2.79	conserved hypothetical protein
BCAL1635	3.07	putative cyclic-di-GMP signaling protein
BCAL1651	2.31	LexA repressor
BCAL1668	2.79	periplasmic solute-binding protein
BCAL1698	2.24	ornibactin biosynthesis protein
BCAL1742	4.50	extracellular solute-binding protein
BCAL1743	2.44	ABC transporter ATP-binding protein
BCAL1744	3.07	binding-protein-dependent transport system inner
BCAL1806	2.73	conserved hypothetical protein
BCAL1844	2.58	conserved hypothetical protein
BCAL1845	2.40	putative membrane protein
BCAL1869	2.07	putative exported protein
BCAL1873	2.83	adenylosuccinate synthetase
BCAL1936	2.09	AhpC/TSA family protein
BCAL1937	2.53	putative phosphorous metabolism-related protein
BCAL2005	2.12	putative membrane protein
BCAL2172	2.79	putative phosphoesterase
BCAL2182	2.34	conserved hypothetical protein
BCAL2204	4.50	IclR family regulatory protein
BCAL2265	3.32	putative membrane protein
BCAL2315	3.69	putative membrane protein
BCAL2375	2.17	putative membrane protein
BCAL2379	3.04	two-component regulatory system, response

Gene name	Fold change	Annotation
BCAL2381	35.59	potassium-transporting ATPase C chain
BCAL2382	31.06	potassium-transporting ATPase B chain
BCAL2383	35.34	potassium-transporting ATPase A chain
BCAL2397	6.67	putative lipoprotein
BCAL2431	2.07	conserved hypothetical protein
BCAL2439	3.60	putative membrane protein
BCAL2450	3.02	putative metal transport integral membrane
BCAL2498	2.06	transposase
BCAL2520	3.13	putative lipoprotein
BCAL2527	2.88	putative hydrolase
BCAL2532	3.57	conserved hypothetical protein
BCAL2533	2.77	putative methionine aminopeptidase (pseudogene)
BCAL2552	2.11	putative membrane protein
BCAL2555	3.00	conserved hypothetical protein
BCAL2561	2.11	LysR family regulatory protein
BCAL2562	2.43	putative integrase (fragment)
BCAL2568	2.11	putative DNA repair protein (fragment)
BCAL2615	2.24	putative exported outer membrane porin protein
BCAL2623	2.07	putative aldehyde dehydrogenase
BCAL2624	2.39	putative aromatic hydrocarbon catabolic
BCAL2625	2.87	Major Facilitator Superfamily protein
BCAL2626	2.46	putative DNA-binding protein
BCAL2692	3.22	LysR family regulatory protein
BCAL2698	3.77	putative membrane protein
BCAL2734	3.61	conserved hypothetical protein
BCAL2795	2.52	aldehyde dehydrogenase family protein
BCAL2796	4.02	putative benzoylformate decarboxylase
BCAL2811	2.66	sorbitol dehydrogenase
BCAL2827	2.25	conserved hypothetical protein
BCAL2833	4.03	putative membrane protein
BCAL2834	2.99	putative acylhydrolase
BCAL2843	4.24	putative branched-chain amino acid transport
BCAL2848	2.66	conserved hypothetical protein
BCAL2931	2.36	radical SAM superfamily protein
BCAL2950	2.88	30S ribosomal protein S1
BCAL2965b	2.03	conserved hypothetical protein
BCAL2980	3.21	putative oxygenase
BCAL3010	2.54	guanosine-3',5'-bis(diphosphate)
BCAL3011	2.91	DNA-directed RNA polymerase omega chain
BCAL3028	2.14	acetyltransferase (GNAT) family protein
BCAL3039	2.29	ABC transporter, membrane permease
BCAL3042	3.34	glucose-6-phosphate 1-dehydrogenase
BCAL3043	2.35	6-phosphogluconolactonase
BCAL3044	2.22	glucokinase
BCAL3104	2.62	urease gamma subunit
BCAL3148	2.63	conserved hypothetical protein

Gene name	Fold change	Annotation
BCAL3176	2.48	AraC family regulatory protein
BCAL3183	3.29	putative hydrolase
BCAL3184	3.22	homogentisate 1,2-dioxygenase
BCAL3185	2.99	4-hydroxybenzoate transporter
BCAL3187	3.07	putative oxidoreductase
BCAL3188	2.05	putative diguanylate phosphodiesterase
BCAL3236	2.06	putative transposase
BCAL3237	2.10	putative transposase
BCAL3252	2.26	putative transposase
BCAL3280	2.57	putative carbon-nitrogen hydrolase protein
BCAL3288	2.97	putative glycolate oxidase subunit GlcD
BCAL3290	2.02	putative glycolate oxidase iron-sulfur subunit
BCAL3295	5.59	putative transposase
BCAL3301	2.08	oxidative stress regulatory protein
BCAL3326	2.25	NAD(P) transhydrogenase subunit alpha
BCAL3339	2.61	holliday junction DNA helicase
BCAL3340	2.11	conserved hypothetical protein
BCAL3406	3.09	putative dehydrogenase
BCAL3409	2.01	IclR family regulatory protein
BCAL3432	2.35	cytochrome c assembly protein
BCAL3454	2.10	conserved hypothetical protein
BCAL3525	2.22	general secretory pathway protein F
BCAM0012	6.45	putative membrane protein
BCAM0013	26.39	putative acetyltransferase - GNAT family
BCAM0023	2.64	acetoacetate decarboxylase
BCAM0025	3.29	putative membrane protein
BCAM0081	2.30	putative outer membrane protein - OmpW family
BCAM0153	3.24	2-keto-3-deoxygluconate permease
BCAM0168	2.04	AraC family regulatory protein
BCAM0176	2.34	AnsC family regulatory protein
BCAM0203	2.23	putative membrane protein
BCAM0289	3.07	two-component regulatory system, sensor kinase
BCAM0394	43.29	conserved hypothetical protein
BCAM0457	2.18	hypothetical protein
BCAM0459	2.51	cysteine desulfurase
BCAM0476a	2.38	hypothetical protein
BCAM0486	2.02	NAD dependent epimerase/dehydratase family
BCAM0514	2.42	TetR family regulatory protein
BCAM0544	76.34	putative acetylglutamate kinase
BCAM0545	114.81	putative PTS transport system
BCAM0545a	13.21	putative membrane protein
BCAM0546	2.20	hypothetical protein
BCAM0547	2.88	putative LysE type translocator
BCAM0568	2.56	putative short chain dehydrogenase
BCAM0620	2.16	Major Facilitator Superfamily protein
BCAM0627	2.46	conserved hypothetical protein

Gene name	Fold change	Annotation
BCAM0629	3.61	putative hydrolase
BCAM0630	4.78	putative dehydrogenase
BCAM0700	2.13	TetR family regulatory protein
BCAM0705	89.29	putative membrane protein
BCAM0727	3.45	conserved hypothetical protein
BCAM0731	3.41	MarR family regulatory protein
BCAM0742	2.25	LysR family regulatory protein
BCAM0757	23.87	putative porin
BCAM0758	41.84	conserved hypothetical protein
BCAM0809	2.09	AraC family regulatory protein
BCAM0870	2.02	putative membrane protein
BCAM0881	6.06	putative alpha amylase-family protein
BCAM0893	3.88	PAP2 superfamily protein
BCAM0897	3.64	MarR family regulatory protein
BCAM0924	3.26	two-component regulatory system, response
BCAM0945	2.88	putative membrane protein
BCAM0997	2.41	HMMPfam hit to PF02674, Colicin V production
BCAM1019	2.38	formate dehydrogenase, iron-sulfur subunit
BCAM1069	2.05	hypothetical phage protein
BCAM1070	2.23	hypothetical phage protein
BCAM1071	2.62	hypothetical phage protein
BCAM1080	2.16	hypothetical phage protein
BCAM1098	2.11	NUDIX hydrolase
BCAM1100	3.89	LysR family regulatory protein
BCAM1127	2.21	putative membrane protein
BCAM1128	2.82	putative glycosyl transferase family protein
BCAM1149	2.67	putative lipoprotein
BCAM1152	6.17	Major Facilitator Superfamily protein
BCAM1193	2.03	SpoVT/AbrB family regulatory protein
BCAM1196	2.34	putative methyl-accepting chemotaxis protein
BCAM1232	3.08	LysR family regulatory protein
BCAM1289	2.28	Major Facilitator Superfamily protein
BCAM1335	6.13	glycosyltransferase
BCAM1337	2.05	glycosyltransferase
BCAM1393	2.02	LysR family regulatory protein
BCAM1410	2.29	putative lipoprotein
BCAM1430	2.15	metallo-beta-lactamase superfamily protein
BCAM1435	2.06	putative hydrolase
BCAM1437	2.03	MarR family regulatory protein
BCAM1466	4.50	IclR family regulatory protein
BCAM1467	2.12	periplasmic solute-binding protein
BCAM1506	3.18	putative phospholipid-binding lipoprotein
BCAM1517	2.03	IclR family regulatory protein
BCAM1554	2.70	putative diguanylate cyclase
BCAM1573	3.05	alpha,alpha-trehalose-phosphate synthase
BCAM1668	5.92	conserved hypothetical protein

Gene name	Fold change	Annotation
BCAM1676	15.27	putative nitrite/sulfite reductase
BCAM1677	17.42	conserved hypothetical protein
BCAM1706	2.59	putative membrane protein
BCAM1724	2.09	MarR family regulatory protein
BCAM1728	2.13	LysR family regulatory protein
BCAM1733	3.26	putative membrane protein
BCAM1739	3.34	putative membrane protein
BCAM1768	2.51	conserved hypothetical protein
BCAM1780A	2.16	conserved hypothetical protein
BCAM1796	2.08	LysR family regulatory protein
BCAM1799	3.22	putative prophage protein
BCAM1809	2.11	MerR family regulatory protein
BCAM1822	3.83	putative NAD-dependent glutamate dehydrogenase
BCAM1829	5.26	putative universal stress protein
BCAM1830	9.62	putative exported protein
BCAM1831	6.85	putative cyclase
BCAM1852	2.62	[2Fe-2S]-binding protein
BCAM1927	7.75	putative exported protein
BCAM1951	2.89	TetR family regulatory protein (pseudogene)
BCAM1962A	2.28	conserved hypothetical protein
BCAM1965	2.02	putative hydrolase
BCAM1994	4.29	hypothetical protein
BCAM2011	2.50	conserved hypothetical protein
BCAM2019	2.35	AraC family regulatory protein
BCAM2035	2.52	putative exopolysaccharide biosynthesis protein
BCAM2064	2.04	putative periplasmic trehalase precursor
BCAM2066	2.05	Major Facilitator Superfamily protein
BCAM2081	9.17	conserved hypothetical protein
BCAM2114	3.62	putative hydroxylase
BCAM2115	2.04	putative 4-hydroxyphenylacetate 3-monooxygenase
BCAM2152	8.20	putative membrane protein
BCAM2190	2.27	LysR family regulatory protein
BCAM2196	3.46	putative acyl-CoA dehydrogenase
BCAM2219	2.06	LysR family regulatory protein
BCAM2287	2.57	conserved hypothetical protein
BCAM2289	8.13	conserved hypothetical protein
BCAM2330	2.26	putative membrane protein
BCAM2354	2.49	hybrid two component system kinase-response
BCAM2393	3.07	AraC family regulatory protein
BCAM2401	3.39	putative aspartyl/asparaginyl beta-hydroxylase
BCAM2403	2.66	conserved hypothetical protein
BCAM2413	49.02	putative GNAT family N-acetyltransferase
BCAM2418	5.52	putative haemagglutinin-related autotransporter
BCAM2419	2.31	putative outer membrane protein A precursor
BCAM2420	2.35	conserved hypothetical protein
BCAM2421	2.91	conserved hypothetical protein

Gene name	Fold change	Annotation
BCAM2422	2.35	putative exported protein
BCAM2500	3.46	putative glucarate transporter
BCAM2501	3.02	shikimate 5-dehydrogenase
BCAM2502	4.55	3-dehydroquinate dehydratase
BCAM2511	2.99	putative D-galactarate dehydratase
BCAM2512	18.25	putative 5-dehydro-4-deoxyglucarate dehydratase
BCAM2514	10.76	putative fatty aldehyde dehydrogenase
BCAM2520	2.09	AsnC family regulatory protein
BCAM2539	2.99	AraC family regulatory protein
BCAM2580	7.81	putative tartrate transporter
BCAM2589	2.77	IclR family regulatory protein
BCAM2682	2.33	putative HicB family protein
BCAM2686	2.61	putative membrane protein
BCAM2720	11.79	putative phospholipase C
BCAM2721	2.48	conserved hypothetical protein
BCAM2764	2.89	putative lipase
BCAM2773	2.46	putative DNA-binding protein
BCAM2787	2.10	AsnC family regulatory protein
BCAM2803	3.10	putative stress-induced protein
BCAM2804	2.23	GntR family regulatory protein
BCAS0021	2.00	putative CoA-transferase
BCAS0062	2.15	LysR family regulatory protein
BCAS0068	2.21	putative transposase
BCAS0070	2.39	two-component regulatory system, response
BCAS0180	2.17	conserved hypothetical protein (pseudogene)
BCAS0194	2.22	conserved hypothetical protein
BCAS0235	2.56	two-component regulatory system, response
BCAS0236	4.44	putative haemagglutinin-related autotransporter
BCAS0238A	3.00	conserved hypothetical protein
BCAS0242	3.98	conserved hypothetical protein
BCAS0244	17.92	hypothetical protein
BCAS0245	19.46	30S ribosomal protein S21 3
BCAS0246	21.83	conserved hypothetical protein
BCAS0247	7.04	hypothetical protein
BCAS0262	2.07	putative acetyltransferase
BCAS0295	2.06	glycosyltransferase
BCAS0321b	2.16	hypothetical protein
BCAS0375	2.49	putative aminotransferase
BCAS0385	2.11	putative acetyltransferase, GNAT family
BCAS0509	2.03	putative phage baseplate assembly protein gpV
BCAS0580	2.12	conserved hypothetical protein
BCAS0581	2.70	putative transcriptional regulatory protein
BCAS0599	2.12	putative phytanoyl-CoA dioxygenase family
BCAS0603	2.19	AraC family regulatory protein
BCAS0613	2.26	AraC family regulatory protein
BCAS0642_J_0	3.46	putative hydrolase (fragment)

Gene name	Fold change	Annotation
BCAS0642_J_1	2.72	putative hydrolase (fragment)
BCAS0646_J_1	3.13	LysR family regulatory protein (pseudogene)
BCAS0684_J_0	4.46	conserved hypothetical protein (fragment)
BCAS0688	5.46	TetR family regulatory protein
BCAS0721	2.23	conserved hypothetical protein
BCAS0722	2.11	putative patatin-like phospholipase
BCAS0757	2.96	conserved hypothetical protein
BCAS0758	2.07	conserved hypothetical protein
pBCA006	2.38	hypothetical protein
pBCA008	3.24	hypothetical protein
pBCA022	2.15	conserved hypothetical protein
pBCA029	2.67	putative membrane protein
IG1_1020850	2.12	interG_chr1_pos_561_1020850:1022405
IG1_1241336	3.25	interG_chr1_pos_682_1241336:1241457
IG1_1303825	2.29	interG_chr1_pos_705_1303825:1311830
IG1_1435033	2.15	interG_chr1_pos_779_1435033:1437975
IG1_1598102	2.10	interG_chr1_pos_869_1598102:1602124
IG1_1633234	2.77	interG_chr1_pos_883_1633234:1633401
IG1_1650963	2.34	interG_chr1_pos_896_1650963:1651093
IG1_1766538	2.11	interG_chr1_pos_953_1766538:1772405
IG1_2151274	2.47	interG_chr1_pos_1124_2151274:2151732
IG1_2206717	2.82	interG_chr1_pos_1141_2206717:2206778
IG1_2379897	3.22	interG_chr1_pos_1211_2379897:2382130
IG1_2434003	2.31	interG_chr1_pos_1226_2434003:2440929
IG1_247826	2.01	interG_chr1_pos_123_247826:247889
IG1_2510004	6.41	interG_chr1_pos_1248_2510004:2511399
IG1_2570370	2.19	interG_chr1_pos_1273_2570370:2570511
IG1_2575094	2.27	interG_chr1_pos_1276_2575094:2575991
IG1_2620209	2.07	interG_chr1_pos_1288_2620209:2620409
IG1_2653432	10.29	interG_chr1_pos_1304_2653432:2653684
IG1_2657870	2.39	interG_chr1_pos_1308_2657870:2669736
IG1_2696183	2.08	interG_chr1_pos_1322_2696183:2696350
IG1_2896142	2.39	interG_chr1_pos_1426_2896142:2897235
IG1_2941513	2.29	interG_chr1_pos_1447_2941513:2941640
IG1_3000663	3.66	interG_chr1_pos_1474_3000663:3003030
IG1_3169576	2.88	interG_chr1_pos_1531_3169576:3169744
IG1_3275858	2.04	interG_chr1_pos_1595_3275858:3276482
IG1_335692	2.21	interG_chr1_pos_205_335692:335775
IG1_3409048	2.15	interG_chr1_pos_1676_3409048:3414161
IG1_3543041	2.17	interG_chr1_pos_1726_3543041:3543103
IG1_3594428	3.92	interG_chr1_pos_1743_3594428:3594489
IG1_360976	2.10	interG_chr1_pos_238_360976:362112
IG1_3752315	2.44	interG_chr1_pos_1813_3752315:3760564
IG1_427870	2.19	interG_chr1_pos_274_427870:435476
IG1_486377	2.15	interG_chr1_pos_301_486377:487637

Gene name	Fold change	Annotation
IG1_489810	5.81	interG_chr1_pos_302_489810:490004
IG1_680158	3.91	interG_chr1_pos_393_680158:681471
IG1_721602	2.48	interG_chr1_pos_412_721602:732050
IG1_845294	2.18	interG_chr1_pos_481_845294:845594
IG1_846330	6.37	interG_chr1_pos_482_846330:846415
IG1_851142	3.92	interG_chr1_pos_485_851142:851227
IG1_958289	3.22	interG_chr1_pos_536_958289:958488
IG1_963484	2.14	interG_chr1_pos_539_963484:963553
IG2_1103454	2.74	interG_chr2_pos_571_1103454:1103560
IG2_1754913	2.45	interG_chr2_pos_896_1754913:1757611
IG2_1823152	2.01	interG_chr2_pos_918_1823152:1823601
IG2_1853918	3.25	interG_chr2_pos_929_1853918:1854189
IG2_1882664	2.11	interG_chr2_pos_938_1882664:1882783
IG2_1926503	22.32	interG_chr2_pos_955_1926503:1926918
IG2_1946878	2.52	interG_chr2_pos_962_1946878:1947020
IG2_2050164	23.26	interG_chr2_pos_1019_2050164:2050639
IG2_2447127	2.79	interG_chr2_pos_1188_2447127:2448873
IG2_2466870	2.82	interG_chr2_pos_1196_2466870:2467109
IG2_2569405	2.28	interG_chr2_pos_1232_2569405:2569553
IG2_2570553	2.02	interG_chr2_pos_1233_2570553:2570841
IG2_2586922	3.16	interG_chr2_pos_1237_2586922:2587412
IG2_2731412	3.33	interG_chr2_pos_1296_2731412:2731800
IG2_3145916	3.57	interG_chr2_pos_1457_3145916:3148349
IG2_3182469	2.11	interG_chr2_pos_1467_3182469:3187274
IG2_321816	2.30	interG_chr2_pos_144_321816:323991
IG2_551370	4.37	interG_chr2_pos_283_551370:553896
IG2_671659	2.91	interG_chr2_pos_350_671659:674180
IG2_683264	4.67	interG_chr2_pos_355_683264:683909
IG2_690307	2.54	interG_chr2_pos_360_690307:693446
IG2_695994	2.20	interG_chr2_pos_362_695994:702120
IG2_794541	2.25	interG_chr2_pos_404_794541:797119
IG3_264432	2.19	interG_chr3_pos_143_264432:264707
IG3_278948	2.24	interG_chr3_pos_148_278948:280854
IG3_811123	2.66	interG_chr3_pos_363_811123:815031
BCAM0269	2.44	intergenic region between BCAM0268 and BCAM0270
BCALr0217b	2.87	tRNA Ile anticodon GAT, Cove score 93.29
BCALr2125f	3.24	tRNA Glu anticodon TTC, Cove score 60.10
BCAMr0727	3.56	tRNA Pseudo anticodon GAA, Cove score 36.58
