# Supplementary material for: Transcriptional response of Burkholderia cenocepacia J2315 sessile cells to treatments with high doses of hydrogen peroxide and sodium hypochlorite
Source: BMC Genomics. 2010 Feb 5;11:90. doi: 10.1186/1471-2164-11-90 (PMC2830190; doi:10.1186/1471-2164-11-90)
Supplement: Additional file 5 — FW and RV primers used in the qPCR experiments. [file 1471-2164-11-90-S5.PDF]

Gene	Annotation	FW primer	RV primer
BCAL0771	Non-heme chloroperoxidase	GCGAAAGCCCTTGTTCACA	CGATGCCAAGGTCATCCATT
BCAL0787	RNA polymerase σ-32 factor	ATCGCCTGCATGCTCTTCTT	ACGAGTACATCCTGCGCAACT
BCAL0953	Recombinase A (*recA*)	GTCTCGATATCCGCCGGATT	GACACCTTGTTCTTGACGACCTT
BCAL1106	Cytochrome b561 family protein	CGATCTATCCGTCGTTCACGTT	CGTCACGGGCAGCAACTT
BCAL1688	RNA polymerase σ^{70} factor (*orbS*)	AGCCGGTCGCCTATGTGA	TCGTCCTCTTCCGTGTGATAGA
BCAL1763	Putative exported protein	GCCTGGATATTGTCCGGATAGTT	CCGGCTTTTGCAAGTGAAA
BCAL1764	Putative exported protein	TCAATGCGGCCTGCAGAT	AGTTGGCACAAGCCGGTTAT
BCAL1765	Putative exported protein	TGCAGATCGTTCGGGTAGGT	AACTCGCGCAGCTTCAACA
BCAL1766	Osm-C family protein (Ohr protein, see text)	ACAGTTGCTCCGGGTTGGT	GGGCAAGACGCATACGACTT
BCAL2014	Carboxymuconolactone family protein	ACAAGCGCAAGTTCGAGATGTAC	CAGCGCATAGTGCGACTTCA
BCAL2297	Conserved hypothetical protein	CGGGTCGTCAACAGCGATA	CGCAACTGATAAGTCTCTCCGTTA
BCAL2780	Putative thioredoxin protein	CCCGTGCTGGTCGACTTCT	TTGACCTTCACGAGCTTCCA
BCAL3299	Catalase (*katB*)	GCTCCCACTCGTAGCTGAACA	AAGGGCAAGGACACGATCA
BCAL3477	Putative catalase	GAACGGCGCGACCGAATA	ACCAACAGCGGCGTCCAT
BCAL3478	Putative RNA polymerase σ factor	CACGATCCTGTACCGACAATTTCT	GTCCGAGAGCCGACCGAAT
BCAM0405	Trans aconitate methyl transferase	GGCACTGCGTTTCGACCTT	GCCGGTTCATCGAGGTTGT
BCAM0740	Conserved hypothetical protein	GCCGACAACGATCACGAGAT	ACCCCCAGACCTTCTCGAA
BCAM0896	Organic hydroperoxide resistance protein	GTATCGGCCGGCAGTGTCT	CGAATCCGGAGCAACTGTTT
BCAM0931	Catalase precursor	TGCCCTGCTGCGTATAGAACT	GACATCTCCGACCTGACGAAA
BCAM1216	Alkyl hydroperoxide reductase F subunit (*ahpF*)	GACTCGACTTCATCCTGGAACAGT	CGACTACGCATTCGAAACGTT
BCAM1217	Alkyl hydroperoxide reductase C subunit (*ahpC*)	CGTAGATTTCGACACCCAGCTT	GAGAACTTCAAGGGCAAATGGT
BCAM1335	Glycosyltransferase	TGTTCGACGAAATCCTGTGT	GCCCTTGTCCTCTTCGATAC
BCAM1554	Putative diguanylate cyclase	GACGCCGCCCTCACCTA	CGGCGTATCGAGCATGATCT
BCAM1833	Aconitate hydratase (*acnB)*	CCGATCTTCTACAACACGATGGA	CGTCGGACTTGACCTGGAAT

Gene	Annotation	FW primer	RV primer
BCAM2107	Catalase (*katA*)	CAACGTGAGTCTCGACAAAGCT	CACCCGTGAGAATCAGCAGAT
BCAS0084	TetR regulatory protein	ATGATGCAGCGCGGTTACA	AGTAGTAGTGGATGCTCGACGTCTT
BCAS0085	Organic hydroperoxide resistance protein	CGAACCCCGAGCAACTGTT	CAGCGAGATCTTCAGTTCGACTT
BCAS0086	Putative exported lipase	CGACTTCAACACCGGTTCGTA	TATAGGCGTCCCAGAACCACTT
BCAS0186	Acyl carrier protein	GCAGCGGACAAGTTCCTGTT	AACACATCCTTCTGCGAGATCA
BCAS0543	Phage transcriptional regulator	TCCTGGAGGTTCTCAACGTTCT	GTTCGTCGCCTTGGTGAGTT
BCAS0546	Rve transposase	GAGTTCTACAAGAACAAGCCAAAGAA	TTCCGCACCCATGACGTACT
BCAS0547	DNA-binding phage protein	CGAACAGCAATACCGATCTCAA	CTTCGTGCGACTGGATGTCAT
BCAL2667	Cell division protein ZapA	GATTCTCGGTCAGCCCTATC	AGCTGTTCGAGCGGATTTT
IG1_2935724	IG between BCAL2667 and BCAL2668	TATTCCTTGAACCAATGCCATGT	TGGTCGCAGTTAGCAGCACTT
BCAL2668	Hypothetical protein	ACTGGCTGATGAAGTCCGA	GGGCAGCTCGAGTGATAGA
BCAL2737-IG1_3008003	Part of putative pseudouridine synthase	CACTCGATATATTCGCGCTC	CAATGCCGGATAGCTAAGGT
IG1_3008003	IG between BCAL2737 and BCAL2738	GCGTCTGTCAACCGAAAGGT	TGTTCATGTTGTGTTCCTCAGCTT
BCAL2738	Hypothetical protein	GTGCACTGCTTCTGGATGA	CTCATTTCGAGCGCATAGAC
