# Supplementary material for: Transcriptional response of Burkholderia cenocepacia J2315 sessile cells to treatments with high doses of hydrogen peroxide and sodium hypochlorite
Source: BMC Genomics. 2010 Feb 5;11:90. doi: 10.1186/1471-2164-11-90 (PMC2830190; doi:10.1186/1471-2164-11-90)
Supplement: Additional file 6 — FW and RV primers used in the qPCR experiments for ten selected reference genes. Five genes encoding functionally diverse proteins were selected based on their moderate and stable expression under all test conditions in the microarray experiments. The other selected candidate reference genes are five of the housekeeping genes used in the B. cepacia complex MLST scheme (in italic) [69]. Based on the pairwise variation analysis (GeNorm) [70], five genes (in bold) were selected for the normalization of the qPCR data. [file 1471-2164-11-90-S6.PDF]

| **Gene** | **Annotation** | **FW primer** | **RV primer** |
|---|---|---|---|
| *BCAL0036* | ATP synthase beta chain | CAAGACCGTCAACATGATGGA | TCGAGTCCTTCATTTCGTGGTA |
| *BCAL0289* | Glutamate synthase large subunit | ATCATCCAGCAGGGTCTGAAGA | GCCATTTCCTCGCGATAGAA |
| *BCAL0421* | DNA gyrase subunit B | GTTCCACTGCATCGCGACTT | GGGCTTCGTCGAATTCATCA |
| ***BCAL1861*** | Acetoacetyl-CoA reductase | GATCACCTGCTTCGTGACGTT | GACGTCGTGTTCCGCAAGAT |
| *BCAM0991* | Tryptophane synthase beta chain | GCCAACGTCTACCGGATGAA | GACCGTGCCGATGATGTAGAA |
| **BCAL1459** | Calcineurin-like phosphoesterase | ATCCCTTGAAATCGAGCATCA | TACGCTCGACAACGTGCATTA |
| **BCAL1659** | Ribose transport permease | ACCGTGTTCGGACGCTATCT | CATCAGTGCGAATACGAGAATTTT |
| **BCAL2694** | Putative dehydrogenase | CTTGCCGTGATCCTCGAGAT | GAGATCAGCGAGGCCGAGTA |
| BCAM2784 | Putative aminotransferase | CCCCGTTCTCGCTCTACGT | GTGTCGCCGAGGCAGAAAT |
| **BCAS0175** | Putative hydrolase | ATGGCCAGTTCGCTCATCA | ACGCGATGTCGATACTCGAAT |
